# Placebo Response in Trials of Negative Symptoms in Schizophrenia: A Critical Reassessment of the Evidence

**DOI:** 10.1093/schbul/sbac061

**Published:** 2022-06-17

**Authors:** Pál Czobor, Brigitta Kakuszi, István Bitter

**Affiliations:** Department of Psychiatry and Psychotherapy, Semmelweis University, Budapest, Hungary; Department of Psychiatry and Psychotherapy, Semmelweis University, Budapest, Hungary; Department of Psychiatry and Psychotherapy, Semmelweis University, Budapest, Hungary

**Keywords:** negative symptoms, schizophrenia, placebo response, predominant negative symptoms, prominent negative symptoms

## Abstract

**Background:**

Summarizing evidence from clinical trials of patients with schizophrenia with predominant or prominent negative symptoms (NS), a prior meta-analysis reported a large placebo effect in negative symptoms (Cohen’s *d* = 2.909). Assuming that such an effect was clinically not plausible, we performed a critical re-assessment and an update of the previous results with newly available data from add-on and monotherapy studies.

**Study Design:**

Random-effect meta/regression analysis of trials that focused on predominant or prominent NS; and adopted a double-blind, randomized, placebo-controlled design. The final pooled meta-analytic database, based on the available add-on and monotherapy studies combined, included 24 publications containing data on a total of 25 studies (21 add-on, 4 monotherapy).

**Study Results:**

The pooled overall estimate for the placebo effect from the primary analysis for all included studies had a medium effect size, with a Cohen’s *d* value of 0.6444 (SE = 0.091). The estimates were similar in the add-on and monotherapy studies. Meta-regression indicated that the high placebo response was significantly associated with clinical trial characteristics, including the high ratio of patients assigned to active vs. placebo treatment and short trial duration.

**Conclusions:**

These results represent a major downward correction for a current effect size estimate of the placebo response in the negative symptoms of schizophrenia. Our findings also pinpoint certain clinical trial characteristics, which may serve as important predictors of the placebo response. The knowledge of these factors can have important implications for drug development and trial design for new drugs for negative symptoms of schizophrenia.

## Introduction

Despite major efforts to develop efficacious treatments, current antipsychotics (APs) have limited efficacy against the negative symptoms of schizophrenia. Negative symptoms (NS) of schizophrenia (eg, blunted affect, anhedonia, avolition, asociality and alogia) consist of two major classes with heterogeneous etiologies, namely the primary and secondary NS.^1^ The primary NS are viewed as an intrinsic part of the schizophrenic illness, and are considered to be associated with specific disease course, and long-term morbidity.^1^ By contrast, the secondary NS are associated with well-defined etiologies, eg, positive symptoms, comorbid depression, side effects of medications, or social deprivation.^1–4^ The treatment of primary NS represents a major unmet need. They may be present before the onset of the first positive symptoms of schizophrenia and persist after the acute phase of the illness, and are associated with poor psychosocial functioning.^1,2,5^ To approve a specifically targeted, domain-specific approach for the development of new drugs for the treatment of negative symptoms, regulators asked an important question: “Do negative symptoms respond differently to drug treatment than other schizophrenic symptoms?” ^6^

With accumulating evidence that available APs provide little benefit for NS, the domain-specific approach was endorsed by the US Food and Drug Administration (FDA) agency in the early 2000s.^6,7^ Furthermore, a consensus between academic experts, regulators and industry representatives was also reached that clinical trials for NS need to develop their specific methodology requirements in order to implement this approach in practice.^8^ Specifically, trials of NS were expected to adopt specific sampling strategies (eg, selection of patients with stable and persistent prominent and predominant NS); inclusion criteria (eg, low severity in other symptom domains, such as positive, depressive, and extrapyramidal symptoms, and cognitive impairment to avoid pseudospecificity); sufficient duration to capture potential improvement; and validated measures of outcome (ie, specific measures of NS, applied as primary endpoint).^8^

Additionally, academic and regulatory experts reached consensus on the need of placebo control in clinical trials to ascertain the specificity of improvement in NS during treatment.^7^ In light of this, it is of interest to turn around the question posed by the regulators, and ask: Do negative symptoms respond to placebo in the same way as other symptoms of schizophrenia? If the placebo effect for NS is high in an adequately selected patient population (as in the case of other symptoms of schizophrenia^9–11^), then it would be essential to find out whether certain clinical trial design or patient level prognostic factors exist that would reduce the placebo response. Identification of these factors could improve our ability to detect the effects of promising new agents for NS.

The difficulty of demonstrating a significant effect against placebo in NS trials is highlighted by Krause et al’s meta-analysis,^12^ which examined monotherapy trials of APs approved for schizophrenia by the U.S. FDA and the European Medicines Agency. This meta-analysis focused on patients with predominant or prominent NS, and found that amisulpride (AMI) was “the only antipsychotic that outperformed placebo in the treatment of predominant negative symptoms”.^12^ It is also noteworthy that even for AMI, the evidence against placebo was equivocal, as all positive studies were conducted prior to the 2000s, before the above requirements for NS trials were stipulated (only one study was conducted after 2000, and it yielded negative results). The trials for the three other APs that were tested besides AMI (olanzapine, zotepine, and sulpride) took place after 2000. It is of note that Krause et al’s meta-analysis (which excluded the add-on trials) did not specifically examine the placebo effect, or address whether the failure of APs against placebo was due to a high placebo response.^13^

We found only one meta-analysis for the placebo effect in clinical trials of patients with schizophrenia with predominant or prominent NS. This meta-analysis by Fraguas et al^14^ covered data from an approx. 15 year period (from January 2002 to January 24, 2017). Unlike Krause et al’s study, Fraguas et al’s meta-analysis investigated placebo-controlled add-on trials (ie, monotherapy trials were not included). The test treatments in the included studies were pharmaceutical agents that have not received regulatory approval for the treatment of schizophrenia. Analyzing the data from 18 studies reported in 17 publications, the authors described a “large placebo response” for NS, with an estimated effect size of 2.909 in terms of Cohen’ *d* measure.

There may be several reasons why a placebo effect of the above magnitude is clinically not plausible in NS studies. First, the analyzed studies adopted the consensus criteria for NS trials which emerged during the early 2000s, including the methodologically stringent provisions for patient selection (eg, requirement for stable predominant or prominent NS, and low severity in other symptom domains). It can be expected that such a carefully selected target population, which presents with stable and enduring NS, is unlikely to evidence a large change in symptom severity over time. Second, it can be hypothesized that unspecific effects including psychological factors that may generally underlie placebo response, such as expectancy effects, optimism and cognitive reframing are modest in this mostly treatment resistant sample. Third, the effect size reported by Fraguas et al.^14^ would entail that, in most cases, the patients assigned to placebo would become almost completely asymptomatic with respect to NS within a trial’s time-frame, which is an unlikely clinical outcome.

In view of these reasons, our principal aim in the current study was to re-assess the placebo effect in randomized double-blind NS studies by focusing specifically on the improvement evidenced by patients in the placebo arms. Furthermore, we also wanted to examine whether the placebo response differs in the monotherapy and the add-on trials. Finally, we aimed to delineate the predictors of the placebo response in the NS studies. Our specific goal was to determine how certain clinical trial-design factors, some of which were reported in the previous meta-analysis as predictors of placebo response (number of arms, industry sponsorship), influence the placebo effect based on the updated estimates.

To accomplish these aims, we re-assessed the effect size estimates which were computed for the individual studies by Fraguas et al.^14^ We implemented adjustments for the estimates due to problems with the original effect size calculation (eg, using standard error, instead of SD, for the computation of Cohen’s *d*). Furthermore, we updated the analysis data set with new data by extending the study inclusion period to the past 20 years. We also broadened the scope of the analyses by including placebo-controlled monotherapy studies besides the add-on trials.

## Methods

### Selection of Publications

We searched for studies that met the following criteria: they (1) focused on predominant or prominent NS; (2) adopted a double-blind, randomized placebo-controlled design; and (3) administered the test treatments either in add-on or monotherapy trials. Similar to Fraguas et al., we selected trials that were published starting from 2002, since as stated by these authors “predominant/prominent negative symptoms only became a target of investigation in the early 2000s”.^14^ The end of the selection period for the current investigation lasted until January 24, 2022, which extended the selection period of the prior meta-analysis (which lasted until January 24, 2017) by 5 years. Methodological details of the search strategy are provided in supplementary material, part 1.

### Computation of Effect Sizes

Similar to Fraguas et al, we used the Cohen’s *d* measure to characterize the placebo effect size for NS. The input parameters for the computation were the mean change of the primary measure of efficacy at the end of treatment in the placebo arm relative to the pretreatment baseline (Chg_PBO_); and the within-group standard deviation of the change (SD_Chg_PBO_within_). Cohen’s *d* was computed by dividing the mean change by its SD (ie, Cohen’s *d* = Chg_PBO_/ **SD**_Chg_**PBO**_within_). From 7 of the included publications the above statistics of change (ie, Chg_PBO_, SD_Chg_PBO_within_) could not be retrieved. Nonetheless, these publications provided the mean and the within-group SD at baseline and at the end of treatment; we used these for the effect size calculation. Specifically, Chg_PBO_ in these studies was computed as raw score difference between the baseline and endpoint (ie, Mean_PBO_bas_, Mean_PBO_end_). For the computation of the within-group SD (SD_Chg_PBO_within_), we used the formula for correlated means provided by Cohen,^15^ since pairs of observations from the same subjects (ie, baseline/endpoint) are expected to be correlated. Accordingly, following Cohen,^15^ we applied the formula below:


$$\begin{array}{l} SD_{Chg\_PBO\_within}=SQRT\left(VAR_{PBO\_bas}+VAR_{PBO\_end}-\;2*\;r*\;SD_{PBO\_bas}*\;SD_{PBO\_end}\right), \end{array}$$

where VAR_PBO_bas_ and VAR_PBO_end_ represent, respectively, the within-group variance of the efficacy measure at baseline and endpoint, and *r* denotes the correlation between baseline and endpoint. Since the *r*-value was not reported in the respective studies, we estimated Cohen’s *d* for various *r*-values. First, the *r*-value of .25, which represents a modest association between baseline and endpoint from the same subjects, was adopted for our primary analysis as a conservative assumption. Then, in sensitivity analyses we investigated whether the effect size estimates would remain similar under various alternative assumptions, including the one of higher correlation (*r* = .5) or no correlation (*r* = 0).

### Moderator Variables for the Meta-Regression Analyses

Besides determining the overall effect size for placebo, we conducted meta-regression analyses to delineate potential predictors of placebo response. First, we investigated whether the placebo effect increased with time in NS trials of schizophrenia (similarly to previous trials of broad-spectrum AP agents which indicated increasing placebo response over the years in the overall symptom severity^11,16^). For this analysis, we used publication date as a moderator variable in the meta-regression. Furthermore, we also examined whether industry sponsorship influenced the placebo response. Since industry sponsorship is typically associated with a large sample size, the total study sample size was also introduced as covariate to investigate whether industry sponsorship has a significant contribution after adjusting for this variable.

For further meta-regression analyses, we classified the potential predictor variables into two sets, and examined them in both univariate and multivariate analyses. One of the sets comprised clinical trial design variables, and included trial type (add-on/monotherapy); study duration (which due to the skewed distribution was classified as short-[*≤*8 weeks] or longer-term[>8 weeks]); sample size in the placebo arm; the ratio of the subjects in the test vs. placebo arm; and study site (used as a dichotomous variable [single center/multi-center], since the number of study sites had skewed distribution). In this analysis, we also investigated the interaction between the active vs. placebo ratio and sample size in the placebo arm to examine whether an association between the active vs. placebo ratio and placebo response varies with the number of patients in the placebo sample. The other predictor variable set comprised basic demographic and clinical characteristics including age; illness duration; gender proportion (female%); and baseline severity of NS, expressed as a fraction (%) within the theoretical severity range of the scale. Further information on the statistical analyses is provided in supplementary material, part 2 (Technical Details of the Statistical Analyses).

## Results

### Included Studies

Detailed description of the results of the automatic search and the manual selection process for the inclusion of publications for this meta-analysis is provided in supplementary material, part 3. Overall, the final pooled meta-analytic database, from the add-on and monotherapy studies jointly, includes 25 studies reported in 24 publications. Supplementary material, part 4 shows the list of publications included in the meta-analysis.

Overall, the current investigation extended the former dataset used in Fraguas et al’s meta-analysis from 18 studies (reported in 17 publications) to 25 studies (reported in 25 publications), by adding the 4 monotherapy studies,^17–20^ and 3 further add-on studies.^21–23^ We also note that for one add-on study included in the former meta-analysis^24^ an updated version of the clinical data became available^25^; we used this data in our analyses for the updated estimates. It should also be noted that for a monotherapy study (^19^) in the former meta-analysis the placebo effect size was determined for comparative purposes with the add-on studies. The effect size, however, was erroneously computed as Cohen’s *d* = 3.255 since SE instead of SD was used in the calculation; the corrected value is 0.366.

### Descriptive Data for the Included Studies

Table 1 provides a detailed characterization of the individual studies included in the current meta-analysis. The description of the NS measures and the computational details for the severity of NS at baseline are shown in supplementary material, part 5.

**Table 1.** Clinical Trial Characteristics and Basic Descriptive Demographic Data for the Studies Included in the Current Meta-Analysis

Studies^a^	Included in Prior Meta-analysis	Study-type	N of Weeks	N of Arms	N of Total Sample	N of PBO Arm Sample	N of Sites	Support^b^	Negative Symptom Measure in Primary Analysis	Age (yrs)	Female%	Illness Duration (yrs)	Baseline NEG severity (%)	test drug
Buchanan, 2007	yes	add-on	16	3	157	52	5	a	modif. SANS avg. item score	43.4	n/a	20.2	46	D-cycloserine, (50 mg) and Glycine (60 mg)
Buchanan, 2015	yes	add-on	12	2	54	28	1	a	modif. SANS total	45.9	75.9	25.4	39.4	Rasagiline (1 mg)
Bugarski-Kirola, 2017, DayLyte study	yes	add-on	24	3	597	201	113	i	PANSS NEG factor score (Marder factor)	42.3	34	n/a	49.5	Bitopertine (5 mg) and Bitopertine(10 mg)
Bugarski-Kirola, 2017, FlashLyte study	yes	add-on	24	3	574	190	122	i	PANSS NEG factor score (Marder factor)	38.7	28.6	n/a	47.9	Bitopertine(10 mg) and Bitopertine(20 mg)
Dunajevich, 2017	yes	add-on	12	4	226	74	58	i	NSA 16 total	43.4	32.9	23.5	61.3	AMG 747
Duncan, 2004	yes	add-on	4	2	22	12	1	a	SANS total	54.4	0	28.6	73.7	D-cycloserine (50 mg),
Goff, 2005	yes	add-on	24	2	55	12	3	a	modif. SANS total	47	28.6	22.4	54	D-cycloserine (50 mg)
Hill, 2011	yes	add-on	12	2	28	14	1	a	modif. SANS total	46.5	13.33	19.1	50.9	Folic acid (1 mg)
Hinkelmann, 2013	yes	add-on	4	3	51	16	1	a	PANSS NEG subscale	38.3	43.75	9.7	46.9	Citalopram (30 mg) and Reboxetine (8 mg)
Pierre, 2007	yes	add-on	8	2	20	10	1	a	modif. SANS total	49.8	10	n/a	42.8	Modafinil (200 mg)
Schoemaker, 2014	yes	add-on	12	3	214	62	25	i	modif. SANS total	38.1	34.30	n/a	58.1	Org25935 (4–8 mg/d mg)and Org25935 (12–16 mg/d mg)
Stauffer, 2013	yes	add-on	16	2	164	82	19	i	NSA 16 total	42.8	21.95	17.8	62.1	PGM (40 mg)
Strous, 2003	yes	add-on	6	2	27	12	1	a	modif. SANS total	37.6	75	16.9	43.2	DHEA (100 mg)
Umbricht, 2014	yes	add-on	8	4	235	61	66	i	PANSS NEG factor score (Marder factor)	39	44	12.8	45.0	Bitopertine (10 mg),Bitopertine(30 mg) and Bitopertine (60 mg)
Usall, 2011	yes	add-on	12	2	33	17	2	a	PANSS NEG subscale	62.7	100	37.4	34.8	Raloxifene (60 mg)
Usall, 2016	yes	add-on	24	2	70	32	3	a	PANSS NEG subscale	61.3	100	34.3	37.6	Raloxifene (60 mg)
Weiser, 2012	yes	add-on	16	2	195	98	10	a	modif. SANS total	39.8	28.6	15.3	65.4	D-serine (2 mg)
Strzelecki, 2018^c^	no	add-on	24	2	60	30	1	a	PANSS NEG subscale	40.2	50	11.6	45.5	Sarcosine (2 mg)
Barnes 2016	no	add-on	12	2	62	32	15	a	PANSS NEG subscale	45.1	31	30	44.5	Citalopram (20 mg)
Bugarski-Kirola, 2022	no	add-on	26	2	403	202	83	i	NSA 16 total	26	32	11.7	63.5	Pimavanserin (10-36 mg)
Hosseininasab, 2021	no	add-on	16	2	58	29	1	a	PANSS NEG subscale	47.8	0	18.8	37.6	Nanoncurcumin 160mg/d
Möller, 2004	no	monoth.	8	2	85	41	14	i	PANSS NEG subscale	42.2	48.8	10.6	48.8	Zotepine (avg dose 131mg/d)
Lecrubier, 2006	no	monoth.	26	4	244	34	multiple	i	modif. SANS, summary score	38.2	35.3	15.4	79.0	OLZ (5 or 20 mg/d), AMI (150mg/d)
Davidson, 2017	no	monoth.	12	3	244	79	36	i	PANSS NEG factor (Pentagonal model)	40	44.7	n/a	35.8	MIN-101 (32mg/d or 64mg/d)
Davidson, 2022	no	monoth.	12	3	513	172	61	i	PANSS NEG factor score (Marder factor)	41	38	n/a	40.5	Roluperidone (MIN-101) (32mg/d or 64mg/d)

*Note*: SANS = Scale for the Assessment of Negative Symptoms; PANSS = Positive and Negative Symptoms Scale; NSA 16 = Negative Symptom Assessment-16 scale; PBO = placebo; NEG = negative symptoms; monoth.=monotherapy; modif. SANS = modified version of the original SANS scale; avg. = average; multiple = multiple study centers (N of sites not provided).

^a^Data from 25 studies, reported in 24 publications, were used in the current meta-analysis. Note: One publication (Bugarski-Kirola, 2017) reported data from two studies (DayLyte & FlashLyte).

^b^Support = source of financial support for the study (a=academia, i=industry)

^c^Clinical data for the Polish Sarcosine Study in Schizophrenia (PULSAR) study were extracted for the current meta-analysis from Strzelecki, 2018. As the prior meta-analysis by Fraguas et al (Schizophr. Bull. 45: 57-68) covered a time period before 2018, it extracted data from an earlier report available for the PULSAR study (Strzelecki D et al. Nutrients 2015; 7: 8767-8782).

References for the included studies:

Buchanan RW et al. Am J Psychiatry 2007; 164: 1593-1602.

Buchanan et al. Schizophr Bull 2015; 41: 900-908.

Bugarski-Kirola D et al. Biol Psychiatry 2017; 82: 8-16 (DayLyte Study)

Bugarski-Kirola D et al. Biol Psychiatry 2017; 82: 8-16. (FlashLyte Study)

Dunayevich E et al. Schizophr Res 2017; 182: 90-97.

Duncan EJ et al. Schizophr Res 2004; 71: 239-248.

Goff DC et al. Psychopharmacology (Berl) 2005; 179: 144-150.

Hill M et al. Schizophr Res 2011; 127: 41-45.

Hinkelmann K et al. J Clin Psychopharmacol 2013; 33: 686-690.

Pierre JM et al. J Clin Psychiatry 2007; 68: 705-710.

Schoemaker JH et al. Clin Psychopharmacol 2014; 34: 190-198.

Stauffer VL et al. Schizophr Res 2013; 150: 434-441.

Strous RD et al. Arch Gen Psychiatry 2003; 60: 133-141.

Umbricht D et al. JAMA Psychiatry 2014; 71: 637-646.

Usall J et al. J Clin Psychiatry 2011; 72: 1552-1557.

Usall J et al. Schizophr Bull 2016; 42: 309-317.

Weiser M et al. J Clin Psychiatry 2012; 73: e728-e734.

Strzelecki D et al. Psychiatry Res 2018; 268: 447-453.

Barnes TR et al. Health Technol Assess 2016; 20: 1-46.

Bugarski-Kirola D et al. Lancet Psychiatry 2022; 9: 46-58.

Hosseininasab M et al. J Clin Psychopharmacol 2021; 41: 25-30.

Möller HJ et al. Pharmacopsychiatry 2004; 37: 270-278.

Lecrubier Y et al. Acta Psychiatr Scand 2006; 114: 319-327.

Davidson M et al. Am J Psychiatry 2017; 174: 1195-1202.

Davidson M et al. Schizophr Bull 2022; 48: 609-619.

Regarding clinical trial characteristics, the majority of studies (18 of 25; 72.0%) were multicenter trials with an add-on design (21 of 25; 84%) and >8 weeks of treatment duration (19 of 25; 76.0%). In 10 of 25 (40.0%) studies, an unbalanced randomization scheme was applied, which assigned twice (in 28% of the studies) or three times (in 12% of the studies) as many patients to the test treatment as to placebo. The average sample size in the placebo arms was 63.7 (SD = 62.1); however, as indicated by the median, only half of the studies had a sample size of *≥*34 patients in the placebo arms. The proportion of industry sponsorship was 44.0% (11 of the 25 PBO arms). However, it is of note that while the rate of industry sponsorship was 33.3% in the add-on studies, all monotherapy trials were industry sponsored. Detailed data on the trial features and the basic demographical and clinical characteristics of the study samples are shown in supplementary material, part 6.

### Meta-Analyses

**Fig. 1.**
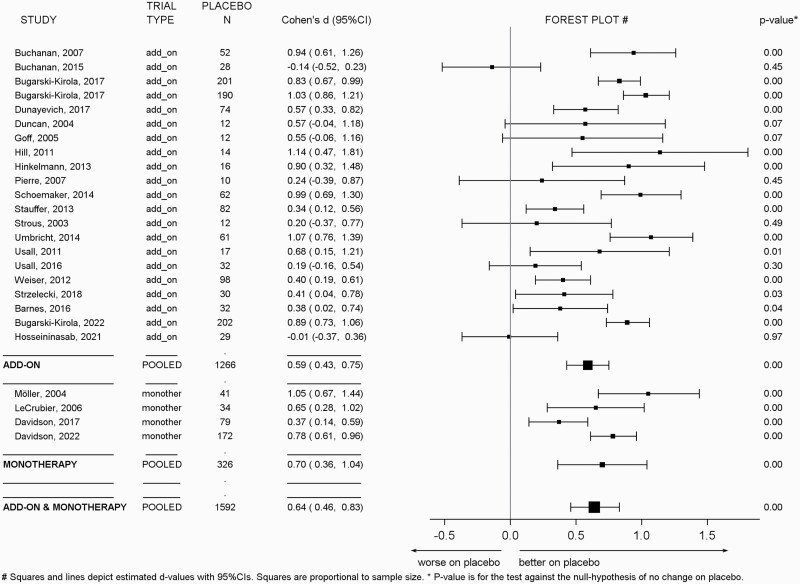
Forest-plot indicating the Cohen’s *d* effect size for the placebo response in the placebo-treated samples in the individual trials of negative symptoms, and for the pooled estimate from the meta-analysis. A random-effect model was used to derive the effect size estimate since the Likelihood Ratio Test (LRT) indicated significant heterogeneity in the effect size estimates across the included studies (LRT = 58.2, df = 1, *P* < .0001). The pooled estimate was determined for all studies and for the add-on and the monotherapy studies, separately. The pooled overall effect size estimate from the primary analysis for all included studies in terms of Cohen’s *d* was 0.6444 (SE = 0.091).The pooled estimates were not significantly different in the add-on (Cohen’s *d* = 0.5898, SE = 0.077) and monotherapy studies (Cohen’s *d* = 0.6993, SE = 0.167). The squares depict the Cohen’s *d* estimates, the horizontal lines indicate the 95% confidence interval of the estimates.

Figure 1 shows the Forest-plot of the effect sizes from the placebo arms of the individual studies and the pooled estimate from the primary analysis. We used a random-effect model to derive the effect size estimate since the Likelihood Ratio Test (LRT) based on the comparison of nested fixed and random-effect models using van Houwelingen et al’s approach^26^ indicated significant heterogeneity in the effect size estimates across the included studies (LRT = 58.2, df = 1, *P* < .0001).

The pooled estimate was computed for all studies and for the add-on and the monotherapy studies, separately. As the plot indicates, all but two of the individual studies had a numerical improvement in the placebo arm, reaching statistical significance in the majority of cases. The pooled overall effect size estimate from the primary analysis for all included studies in terms of Cohen’s *d* was 0.6444 (SE = 0.091). The pooled estimates were similar for the add-on (Cohen’s *d* = 0.5898, SE = 0.077) and monotherapy studies (Cohen’s *d* = 0.6993, SE = 0.167).

Table 2 provides further details for the effect size estimates, including the results of the sensitivity analyses. These analyses were conducted since not all studies reported the basic summary statistics for the mean change from baseline to endpoint. As shown by the Table, the pooled estimates from the sensitivity analyses showed only little variation. Specifically, considering all included studies, the lowest and the highest pooled effect size was 0.6367 (SE = 0.092) and 0.6566 (SE = 0.091), respectively. For the add-on studies, the lowest and highest estimate from the sensitivity analysis was 0.5742 (SE = 0.078) and 0.6142 (SE = 0.077), respectively. We note that for the monotherapy trials, the sensitivity estimates did not vary as there were no missing data in the respective reports. The analyses did not indicate the presence of publication bias on the basis of the funnel plots using Begg & Mazumdar’s rank correlation approach^27^ and the Egger test^28^ (*P* > .1 in both analyses).

**Table 2.** Placebo Effect Size Estimates From Add-On and Monotherapy Studies of Pharmaceutical Agents in Patients With Predominant or Prominent Negative Symptoms

Studies^a^	Study_type	Current Analysis - Reassessed Estimates								
		Cohen’s *d* Estimate From Primary Analysis (*r* = .25)^c^^,^			Cohen’s *d* Estimate From Sensitivity Analysis (*r* = .0)^c^			Cohen’s *d* Estimate From Sensitivity Analysis (*r* = .50)^c^^,^		
		*d* (SE)	*t*	*P*	*d* (SE)	*t*	*P*	*d* (SE)	*t*	*P*
Buchanan, 2007	add-on	0.936 (0.166)	5.629	.000	0.936 (0.166)	5.629	.000	0.936 (0.166)	5.629	.000
Buchanan, 2017	add-on	-0.143 (0.190)	-0.752	.452	-0.143 (0.190)	-0.752	.452	-0.143 (0.190)	-0.752	.452
Bugarski-Kirola, 2017 DayLyte study^b^	add-on	0.829 (0.082)	10.140	.000	0.829 (0.082)	10.140	.000	0.829 (0.082)	10.140	.000
Bugarski-Kirola, 2017 FlashLyte study^b^	add-on	1.035 (0.090)	11.510	.000	1.035 (0.090)	11.510	.000	1.035 (0.090)	11.510	.000
Dunayevich, 2017	add-on	0.572 (0.125)	4.559	.000	0.572 (0.125)	4.559	.000	0.572 (0.125)	4.559	.000
Duncan,2004	add-on	0.570 (0.311)	1.832	.067	0.494 (0.306)	1.616	.106	0.698 (0.322)	2.169	.030
Goff, 2005	add-on	0.552 (0.310)	1.782	.075	0.480 (0.305)	1.574	.116	0.673 (0.320)	2.105	.035
Hill, 2011	add-on	1.141 (0.343)	3.323	.001	1.141 (0.343)	3.323	.000	1.141 (0.343)	3.323	.001
Hinkelmann, 2013	add-on	0.902 (0.297)	3.043	.002	0.790 (0.286)	2.758	.006	1.083 (0.315)	3.440	.001
Pierre, 2007	add-on	0.243 (0.321)	0.758	.449	0.211 (0.320)	0.660	.510	0.297 (0.323)	0.920	.358
Schoemaker, 2014	add-on	0.994 (0.155)	6.402	.000	0.994 (0.155)	6.402	.000	0.994 (0.155)	6.402	.000
Stauffer, 2013	add-on	0.340 (0.114)	2.994	.003	0.340 (0.114)	2.994	.003	0.340 (0.114)	2.994	.003
Strous, 2003	add-on	0.201 (0.292)	0.689	.491	0.201 (0.292)	0.690	.491	0.201 (0.292)	0.689	.491
Umbricht, 2014	add-on	1.073 (0.161)	6.676	.000	1.073 (0.161)	6.676	.000	1.073 (0.161)	6.676	.000
Usall, 2011	add-on	0.680 (0.269)	2.528	.011	0.680 (0.269)	2.528	.011	0.680 (0.269)	2.528	.011
Usall,2016	add-on	0.186 (0.178)	1.044	.297	0.186 (0.178)	1.044	.297	0.186 (0.178)	1.044	.297
Weiser, 2012	add-on	0.400 (0.105)	3.812	.000	0.347 (0.104)	3.336	.001	0.489 (0.107)	4.576	.000
Strzelecki, 2018	add-on	0.412 (0.190)	2.165	.030	0.412 (0.190)	2.165	.030	0.412 (0.190)	2.165	.030
Barnes, 2016	add-on	0.382 (0.183)	2.083	.037	0.331 (0.182)	1.823	.068	0.465 (0.186)	2.500	.012
Bugarski-Kirola, 2022	add-on	0.893 (0.083)	10.728	.000	0.893 (0.083)	10.728	.000	0.893 (0.083)	10.728	.000
Hosseininasab, 2021	add-on	-0.007 (0.186)	-0.038	.970	-0.006 (0.186)	-0.031	.974	-0.009 (0.186)	-0.047	.963
**Pooled estimate, add-on studies**	**All add-on studies**	**0.5898 (0.077)**	**7.66**	**.000**	**0.5742 (0.078)**	**7.41**	**.000**	**0.6142 (0.077)**	**7.99**	**.000**
Möller, 2004	monother.	1.054 (0.195)	5.410	.000	1.054 (0.195)	5.410	.000	1.054 (0.195)	5.410	.000
Lecrubier, 2006	monother.	0.646 (0.189)	3.426	.001	0.646 (0.189)	3.426	.001	0.646 (0.189)	3.426	.000
Davidson, 2017	monother.	0.366 (0.116)	3.151	.002	0.366 (0.116)	3.151	.002	0.366 (0.116)	3.151	.002
Davidson, 2022	monother.	0.785(0.087)	9.000	.000	0.785 (0.087)	9.000	.000	0.785 (0.087)	9.000	.000
**Pooled estimate, monotherapy**	**All monother. studies**	**0.6993 (0.167)**	**4.20**	**.000**	**0.6993 (0.167)**	**4.20**	**.000**	**0.6993 (0.167)**	**4.20**	**.000**
**Pooled estimate, all combined**	**All add-on & monother.**	**0.6444 (0.091)**	**7.08**	**.000**	**0.6367 (0.092)**	**6.93**	**.000**	**0.6566 (0.091)**	**7.24**	**.000**

*Note*: *r*-value = coefficient of correlation between baseline and endpoint; SE = Standard Error of the Cohen’s *d* estimate; t and *P* indicate the test-statistic and the Type I error, respectively.

^a^Data on 25 studies, reported in 24 publications, were used in the analyses.

^b^Bugarski-Kirola, 2017 reported data from two studies (DayLyte & FlashLyte).

^c^From 7 of the 25 included studies the baseline – endpoint change statistics for the Cohen’s *d* computation could not be retrieved, as 6 studies (Duncan 2004, Goff 2005, Hinkelmann 2013, Pierre 2007, Weiser 2012, Barnes 2016) did not directly report these statistics and in one publication (Hosseininasab, 2021) the values were not clearly identifiable. Nonetheless, each of these publications provided the mean and the within-group SD at baseline and at the end of treatment, respectively. To estimate the effect size, these values were used in the primary and sensitivity analyses, adopting a correlation coefficient (*r*-value) of .25, .0, and .5 between baseline and endpoint, respectively (see Methods for further details). The *r*-value of .25, which represents a modest association between baseline and endpoint from the same subjects, was adopted for the primary analysis. The sensitivity analysis was conducted for the *r*-value of .0 and .5, respectively. Please note that for those 18 studies (ie, 25-7) which directly reported the baseline – endpoint change statistics, no estimation under various sensitivity assumptions had to be conducted (thus, the values for these studies across the respective columns of the table do not change).

Together, our results indicate that the effect size estimate of the placebo response in NS is approximately one-fifth of the estimate reported by the previous meta-analysis (ie, Cohen’s *d* = 2.909).^14^Supplementary material, part 7 details the reasons why the approximately five-fold inflation of the effect size occurred in the previous meta-analysis.

### Meta-Regressions

Table 3 displays the meta-regression findings for the putative predictors of placebo response of NS based on all trials (add-on/monotherapy combined). Regarding trial characteristics, the univariate analyses showed that the ratio of active vs. placebo treated patients and the sample size had a significant positive association with the placebo effect size. With respect to patient demographics, illness duration approached statistical significance (*P* = .061) with an inverse relationship with the placebo response: patients with longer illness duration tended to show lower placebo response. We also found that industry sponsorship and larger trial size were associated with greater response to placebo, while publication date had no association.

**Table 3.** Predictors of Placebo Response: Meta-regression Results

Moderator Variables	Number of Studies^a^	Meta-regressions						
		Univariate Regression Results			Multivariate Analysis Variable Set	Multivariate Regression Results		
		Coefficient (95% CI)^b^	*t*	*P*		Coefficient (95% CI)^b^	*t*	*P*
Publication year	25	-0.004 (-0.030 to 0.022)	-0.32	.753	n/a	n/a -		
Trial characteristics								
Add-on	25	-0.109 (-0.488 to 0.269)	-0.60	.555	1	0.100 (-0.151 to 0.351)	0.89	.395
Multicenter	25	0.237 (-0.102 to 0.576)	1.44	.162	1	0.134 (-0.171 to 0.439)	0.90	.375
Trial duration	25	-0.154 (-0.515 to 0.207)	-0.88	.388	1	**-0.367 (-0.652 to -0.083)**	**-2.65**	**.014**
N of patients in the placebo arm	25	**0.002 (-0.001 to 0.004)**	**2.32**	**.033**	1	0.003 (-0.001 to 0.008)	1.66	.137
Active/PBO ratio	25	**0.228 (0.051 to 0.405)**	**2.70**	**.010**	1	**0.249 (0.001 to 0.005)**	**2.10**	**.049**
Patient characteristics								
Illness duration	19^c^	-0.019 (-0.040 to 0.001)	-1.99	.061	2	-0.035 (-0.075 to 0.005)	-1.86	.079
Age	25	-0.020 (-0.041 to 0.001)	-1.99	.058	2	0.021 (- 0.023 to 0.066)	1.00	.326
Female %	24^c^	-0.003 (-0.009 to 0.004)	-0.85	.403	2	0.002 (-0.005 to 0.009)	0.51	.613
Baseline severity	25	0.007 (-0.006 to 0.019)	1.12	.274	2	0.008 (-0.007 to 0.023)	1.08	.294
Industry sponsorship	25	**0.350 (0.104 to 0.596)**	**2.94**	**.007**	3	0.261 (-0.106 to 0.628)	1.51	.152
Trial size (total *n*)	25	**0.001 (0.000 to 0.002)**	**2.88**	**.011**	3	0.001 (-0.001 to 0.001)	1.09	.299

*Note*:

^a^Data on 25 studies, reported in 24 publications.

^b^Coefficient (95% CI) = regression coefficient from the random-effect meta-regression model; t and *P* indicate the test-statistic and the Type I error, respectively. Significant regressor-effects are highlighted in bold.

^c^ *N* is less than 25 due to missing data.

**Fig. 2.**
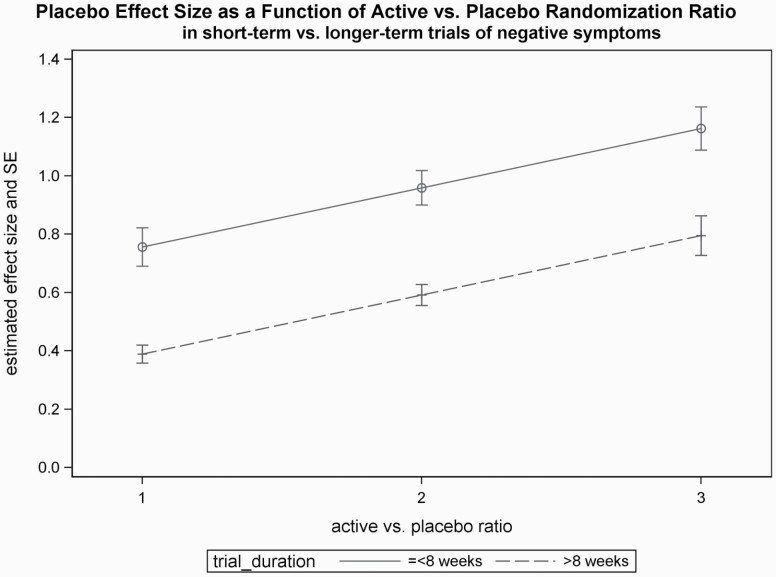
Placebo effect size in negative symptoms as a function of the duration of the trial and of the ratio of patients assigned to active vs. placebo treatments (active vs. placebo ratio) in a given study. The values depicted in the Figure represent Least-Squares Mean estimates from the multivariate random-effect meta-regression analysis; the vertical lines indicate the standard error of the estimates. Numerical results pertaining to the model are shown in Table 3. As shown by the Figure, a considerably smaller placebo effect can be achieved by a combination of longer trial duration (>8 weeks) and a balanced active vs. placebo ratio of treatment assignments (ie, 1:1 ratio).

We also performed multivariate analyses to understand the specific contribution of the variables in the various sets of predictors (labeled as set 1 through 3 in table 3) in determining the placebo response. The results in table 3 show that in the set of trial characteristics included, the ratio of active vs. placebo treated patients and the effect of trial duration reached significance in the multivariate analysis. Shorter trial duration and greater active vs. placebo ratio were associated with larger placebo response. There was no significant interaction between the active vs. placebo ratio and the number of patients in the placebo group (*t* = −0.59, *P* = .571). The joint additive association of placebo response with the ratio of active vs. placebo ratio and trial duration is presented in figure 2, which shows that smaller placebo effects are associated with a combination of longer trial duration (>8 weeks) and a balanced active vs. placebo ratio (ie, 1:1 assignment). We also found that in the set of “patient characteristics” variables included in table 3 (set 2), illness duration remained marginally significant after adjusting for the other variables in the set. Our third analysis showed that, after adjusting for each other’s contribution, in the multivariate analysis neither trial size (ie, total N) nor industry sponsorship was able to serve as an independent predictor, as both of these variables lost significance.

## Discussion

This study provides an update and critical reassessment of a previous meta-analysis of the placebo effect in negative symptoms in schizophrenia.^14^ Two research questions were evaluated: 1) What is the magnitude of the placebo response (the effect size) in patients with predominant or prominent NS? 2) What are the predictors of placebo response in these studies?

### Effect Size Estimation

With respect to the first question, our results indicated a medium effect size (Cohen’s *d* = 0.6444). It is noteworthy that the results showed no difference between the add-on and monotherapy trials; they were numerically almost identical. Overall, our findings represent a major downward correction of the estimate from the previous meta-analysis, which reported approximately five times higher effect size. We found that much of the discrepancy between the prior and current estimates is due to the incorrect computation of the effect size in the previous study. With this in mind, we would like to note that in terms of widely accepted benchmarks (which describe Cohen’s *d* values of 0.2, 0.5, and 0.8, as small, medium, and large, respectively (Cohen^15^)), the placebo effect size estimate (0.6444) that we found for NS can be viewed as being at the higher end of the medium effect size range.

The finding that the placebo effect size estimate is substantially smaller than the cited estimate is consistent with empirical data indicating that negative symptoms, especially the primary negative symptoms demonstrate marked stability during the entire disease course.^1,2,4,13,29,30^ Stable disease course and chronicity have been shown to be associated with lower placebo response rates across various psychiatric illnesses including depression,^31^ while fluctuating course is a predictor of high placebo response, as many episodes tend to be time limited.^32^ With respect to NS studies in schizophrenia, the expert consensus opinion for patient selection criteria stated that “Negative symptoms should be stable and persistent. If negative symptoms fluctuate during a trial, this may increase the proportion of subjects who improve on the control condition.” ^8^ Collectively, our results that show only a medium effect size for placebo in the NS studies can be viewed as a corroboration that the consensus recommendations are reaching their goals clinical trials’ practice. Nonetheless, we should also bear in mind that, despite the recommendations, a substantial variation in the study samples and in the assessment and choice of rating scales for NS can still be present in the randomized clinical trials that can introduce heterogeneity and bias, which adds to the complexity of interpreting changes in negative symptoms.^29,33^

The question can be raised whether the placebo effect size for NS is comparable to the placebo effect size for other symptoms of schizophrenia. Since comparative meta-analytic summaries of the placebo response across various symptoms dimensions of schizophrenia are lacking, this question is currently difficult to address directly. With respect to the placebo effect for overall symptom severity, a large meta-analysis by Agid et al.,^11^ which used the same effect size measure as we used in the current meta-analysis, yielded a Cohen’s *d* value of 0.33. This value is somewhat lower than the placebo effect size we found for the negative symptoms (ie, Cohen’s *d* = 0.6444). However, two important caveats have to be taken into consideration in order to interpret the lower effect size in Agid et al’s study. First, similar to other studies which used raw score based measures to investigate placebo response (eg,^10,34^), Agid at al’s meta-analysis included many studies where a single-blind initial placebo run-in period was used to exclude placebo responders to increase assay-sensitivity. Thus, patients who were likely to evidence a large response to placebo were not included in the double-blind phase of these trials. Second, the fact that the overall symptom severity measures also included negative symptoms precludes a clear conclusion regarding the placebo effect size in NS vs. the rest of symptoms of schizophrenia, which highlights the need for further studies.

### Predictors of Placebo Response (Meta-Regression)

Besides the estimation of the placebo effect for NS, we conducted meta-regression analyses to identify the predictors of the placebo response. Regarding trial characteristics, we found that add-on and monotherapy trials were similar in the magnitude of the placebo effect. Furthermore, with respect to trial characteristics, one of the most robust predictors of placebo response was the active vs. placebo ratio, with congruent results from univariate and multivariate analyses. Specifically, we found that the higher the number of patients assigned to the active group(s) relative to placebo, the greater the placebo response. This finding for the negative symptoms is similar to most other findings reported for the placebo effect for the overall symptom severity in schizophrenia (eg,^11,35^). Thus, our results can be viewed as an extension of the prior results to the negative symptoms of schizophrenia.

Additionally, we note that a consistent picture regarding the predictive role of the active vs. placebo ratio emerges when we compare the findings from schizophrenia trials to the findings from other psychiatric disorders, especially major depression where the role of the active vs. placebo ratio is well-documented.^36,37^ This predictor, therefore, can be conceived of as a general methodological factor across various disease conditions, whose effect is mediated by unspecific factors rather than by the symptoms of the disorder. We think that one of the most likely candidates for such an unspecific predictor can be the expectancy effect, which can enhance treatment effects via expectations from either the patient or the physician through the anticipation that the current treatment is efficacious. Specifically, with higher active vs. placebo ratio a higher proportion of patients receive active treatment, which can increase the overall expectations and thereby results in higher placebo response, which we found in this study.

Study duration also reached significance in the multivariate analyses as a predictor of placebo response. Specifically, we found that the shorter the trial, the larger the placebo response. This finding is also consistent with results from former antipsychotic trials in schizophrenia (see, eg, Leucht et al’s meta-analytic summary^10^). It also highlights that longer trial duration is more suitable to reduce placebo effect, and to adequately capture changes in negative symptoms which are not associated with placebo response. Furthermore, it supports the conclusion of the expert panel that, while briefer duration of treatment of negative symptoms is acceptable for proof-of-concept trials, “pharmacological clinical trials need to be no less than 3 months but optimally 6 month or longer not including a prerandomization phase for the purpose of stabilization”.^8^

We also examined whether industry sponsorship is a predictor of placebo response in NS studies. Because industry sponsorship is often accompanied by larger sample size, we investigated these two variables (industry sponsorship, sample size) both in univariate and multivariate analyses. In the univariate analyses, consistent with former reports we found that both variables were associated with larger placebo response. As to the explanation of the trial size effect, we agree with Leucht et al’s conclusion that “… in studies with large sample size more patients who benefit from placebo are recruited compared to smaller trials where patients can be more carefully selected.” ^10^ We also concur with Leucht et al that while industry-sponsoring tends to increase placebo response, “industry sponsorship probably is a composite of multiple factors”,^10^ including the co-occurrence of larger sample size with the industry sponsored studies. Indeed, when these variables were examined jointly in our multivariate analysis, neither of them could provide a significant independent contribution.

In terms of patient characteristics, our analyses yielded only one marginally significant finding which occurred both in the univariate and the multivariate analysis. Specifically, the results indicated that longer illness duration was associated with lower placebo response independent of the effect of age, while age, in itself, was not associated with the placebo response as indicated by the multivariate analysis (*P* > .1). These findings for the NS, while only approached statistical significance, are congruent with the results of several meta-analyses which focused on the overall symptom severity, and showed a negative association with the illness duration.^10,11^ It is important to note that the inverse relationship between illness duration and placebo response is not specific to schizophrenia; chronicity is associated with lower placebo response rates in other psychiatric illnesses as well (eg, depression^31^). An explanation for the association in schizophrenia was put forward by Agid et al who stated that patients who have “shorter duration of illness may be more susceptible to placebo response because of heightened expectations based on treatment earlier in the course of illness.“ ^11^ Nonetheless, it is important to note that with respect to NS, the current study identified only a potential signal that illness duration can serve as a predictor of placebo response; this requires confirmation.

We found no change in the placebo response with calendar time, which is noteworthy in the context of several reports on an increasing placebo response over the years.^9,11,16^ It should be noted, however, that in some of the studies the time effect was not present^35^ and that the meta-analyses that reported increasing placebo response over time typically span longer than the two decades^11^ period, which we focused on in the current meta-analysis. One also needs to consider that the calendar time effect on placebo response is closely related to changes in trial design factors, eg, increase in the number of subjects and sites, and change in treatment settings over time.^11^ These factors remained relatively stable during the period that we examined, since the required trial design features were stipulated in the early 2000s for the NS studies.

Our study has several limitations. First, the small number of monotherapy trials did not make possible a detailed analysis of these trials. Nonetheless, the pooled effect size for these trials did not show a considerable deviation from the pooled effect size of the add-on trials, indicating that the lack of significant difference was not due the lack of statistical power, but to the rather small difference between the two types of studies. Second, some of the included studies did not report the difference scores from baseline to endpoint, but instead reported the raw scores and standard deviations at the baseline and the end point, separately. Thus, the effect size measures from these studies were estimated on the basis of various assumptions on the correlations between the baseline and endpoint scores. We conducted sensitivity analyses to examine the impact of these assumptions, and found that the results did not exhibit a substantial change as a function of the various assumptions. Furthermore, we note that of some of the information on patient characteristics was missing from certain studies. However, since the proportion of missing data was rather small, we think that our regression estimates are robust with respect to the missing data.

Finally, it should also be noted that, due to several potential biases that occur in the clinical trial environment, the medium placebo effect size that we found for NS can still be an overestimate of the “true” placebo effect. For example, it is noteworthy that despite the strict inclusion criteria for the NS studies, and the low level of symptom fluctuations in patients with primary NS, the regression to the mean effect can still be operant since patients with high baseline severity are enrolled in these studies, who can spontaneously improve over time. It is also conceivable that some of the clinical trials might include patients with transient (secondary) negative symptoms besides their primary negative symptoms (or even patients without primary negative symptoms). Furthermore, because the severity of NS is required to exceed a certain threshold, baseline inflation can also occur due to clinician bias to enroll subjects in the study, which may lead to increased placebo response.^38^ Study participants could also evidence the clinical trial (Hawthorne) effect, which can be mediated through various factors.^39,40^ These can include the impact of receiving more attention and being better observed in a clinical study, slightly better care during the trial, and selection bias from the physicians to work with more adherent patients.

Overall, our update and critical reassessment of the previous meta-analysis of the placebo effect on negative symptoms in schizophrenia revealed a medium effect (Cohen’s *d* = 0.6444) for the placebo response in the negative symptoms of schizophrenia. This represents a major downward adjustment compared to the previous report. In terms of meta-regression results, the current study uncovered that several study design factors have a profound impact on the magnitude of the placebo response. For example, a longer-term trial with a balanced active placebo ratio is expected to produce an effect size of 0.389 (SE = 0.0619); by contrast a short-term trial with unbalanced active vs. placebo ratio is associated with a placebo effect size of 1.162 (SE = 0.1483). The knowledge of these factors has important implications for drug developers: it can help reducing the placebo response with a suitable study design, thereby increasing the assay-sensitivity to detect the effects of promising new agents for negative symptoms.

## Supplementary Material

sbac061_suppl_Supplementary_Material
