# Supplementary material for: Placebo Response in Trials of Negative Symptoms in Schizophrenia: A Critical Reassessment of the Evidence
Source: Schizophr Bull. 2022 Jun 17;48(6):1228–40. doi: 10.1093/schbul/sbac061 (PMC9673255; doi:10.1093/schbul/sbac061)
Supplement: sbac061_suppl_Supplementary_Material [file sbac061_suppl_supplementary_material.pdf]

ONLINE Supplement

Table of Contents

ONLINE SUPPLEMENT, PART 1: DETAILS OF THE SEARCH STATEGY 2

ONLINE SUPPLEMENT, PART 2. TECHNICAL DETAILS OF THE STATISTICAL ANALYSES. 3

ONLINE SUPPLEMENT, PART 3. RESULTS OF THE AUTOMATIC SEARCH AND THE MANUAL SELECTION PROCESS FOR THE INCLUSION OF PUBLICATIONS IN THE DATABASE. 4

ONLINE SUPPLEMENT, PART 4. LIST OF THE FINAL SET OF PUBLICATIONS INCLUDED IN THE META-ANALYSIS. 11

ONLINE SUPPLEMENT, PART 5. DESCRIPTION OF THE NEGATIVE SYMPTOM MEASURES USED IN EACH STUDY & THE COMPUTATIONAL DETAILS FOR THE SEVERITY OF NEGATIVE SYMPTOMS AT BASELINE. 13

ONLINE SUPPLEMENT, PART 6. BASIC DEMOGRAPHICAL AND CLINICAL CHARACTERISTICS OF THE STUDY SAMPLE INCLUDED IN THE PUBLICATIONS. 16

ONLINE SUPPLEMENT, PART 7. SPECIFIC REASONS OF REASSESSMENT OF EFFECT SIZE ESTIMATES FROM PREVIOUS META-ANALYSIS. 17

Online Supplement, Part 1: Details of the Search Stategy

The current meta-analysis focused on studies that were published in English. We formulated three query expressions (Q1-Q3) to select studies from the Pubmed and Web of Science databases according to the above criteria. Our search targeted all available fields in the bibliography database, including (but not limited to) the key words, the title and the abstract. The first two query expressions (Q1, Q2) were the same for the add-on and the monotherapy studies, whereas the third one differed depending on whether add-on (Q3a) or monotherapy studies Q3m) were selected for the analysis.

Specifically, the first query expression (Q1) focused on the selection of studies with predominant or prominent negative symptoms. It included a number of potentially relevant search phrases (see in quotation marks below) which were connected with logical operators (AND, OR) in the following form:

> Q1 = ("negative symptoms" AND (predom* OR "severe negative" OR "persistent negative" OR "prominent negative" OR prominent* OR "persisting negative" OR "enduring negative" OR "dominant negative" OR ("deficit syndrome")) AND (2002:2022[pdat]))
>
> (Please note that the character * was used as a wildcard.)

The second query expression (Q2) that we used for the selection of randomized, double-blind placebo controlled (DBRPCT) studies was the following:

> Q2 = ("placebo" AND ("double-blind placebo-controlled" OR "double-blind controlled" OR ("double-blind")) AND (2002:2022[pdat]))

The third query expression (Q3) was applied to select, respectively, studies with either an add-on (Q3a) or a monotherapy design (Q3m).

> For the add-on studies, we applied the following expression:
>
> Q3a = ("add-on" OR "augmentation" OR "adjunctive" OR "supplement" OR "supplementation" OR "adjuvant" AND (2002:2022[pdat]))
>
> For the monotherapy studies, the search expression was the following:
>
> Q3m = NOT "add-on" NOT "augmentation" NOT "adjunctive" NOT "supplement" NOT "supplementation" NOT "adjuvant"

Because all three conditions (negative symptoms, DBRPCT, and add-on or monotherapy setting) had to be met in order to be included in the analysis, for our final selection of the publications the three query conditions were connected by the logical operator AND using the following form.

> For the add-on studies: Qadd-on = Q1 AND Q2 AND Q3a.
>
> For the monotherapy studies: Qmono = Q1 AND Q2 AND Q3m.

On the basis of the results of the automatic search, two of the authors (PC, BK) identified potentially relevant publications, and obtained the published reports for further evaluation. The same two authors independently evaluated the pre-selected articles for their eligibility for inclusion according to the previously defined inclusion criteria. In case any doubt arose, the uncertainties were resolved through a

consensus discussion with the third member (IB) of the research group. In addition to the automatic search, we also checked the references of relevant publications for additional reports not identified by the database search. At the conclusion of the search process, the search results from the add-on and monotherapy queries were pooled into a single database. From the final pooled set of selected publications, we extracted the data onto standard, simple forms, and converted them into a Statistical Analysis System (SAS) 9.4 database.

Online Supplement, Part 2. Technical Details of the Statistical Analyses.

Statistical Analyses

The pooled effect size for the placebo effect (i.e., change from baseline to endpoint in the placebo arms) was estimated using the random-effect model for meta-analysis (Van Houwelingen, Arends, & Stijnen, 2002). The random-effect model was chosen because heterogeneity, regardless of statistical significance, is likely to be present in trials that have marked differences in design and study populations. The analysis under heterogeneity applied the DerSimonian and Laird (DerSimonian & Laird, 1986) normal mixture model, which considers each sample as an independent random sample from a normal population. First, the pooled effect size for the add-on and the monotherapy trials was estimated in the meta-analysis, which included trial design (add-on vs. monotherapy) analysis as a factor. Subsequently, the data from the two types of trials were pooled and examined jointly since the estimates from the add-on and monotherapy trials were essentially the same. Trial type was added as covariate in the pooled analyses. The meta-analytic effect-size estimates can be distorted by a tendency of not publishing negative or inconclusive studies in the literature. The possibility of publication bias in our meta-analysis was investigated on the basis of funnel plots using Begg & Mazumdar's rank correlation approach (Begg & Mazumdar, 1994); and the Egger test (Egger, Davey, Schneider, & Minder, 1997). In the Egger test, the standard normal ES is regressed on its precision, defined as the inverse of the standard error(Egger et al., 1997).

The meta-regression analysis was based on van Houwelingen et al.'s general linear mixed model (GLMM) approach using the approximate likelihood method (Van Houwelingen et al., 2002). In the GLMM, the effect size from each study was regressed on an intercept and on the study-level moderator variables from the individual studies (described in the previous subsection). The GLMM model incorporated both fixed and random effects. Based on the random effect part of the model, a common weighted statistical meta-regression effect-size estimate was calculated through the DerSimonian–Laird estimator (DerSimonian & Laird, 1986). The meta-regressions examined the moderators both in univariate and multivariate analyses. The meta-regression analysis was performed for all studies (i.e., for the add-on and monotherapy trials combined). A separate meta-regression by trial type was not performed since the number of monotherapy trials was too low (n=4). All analyses were conducted using the SAS statistical software version 9.4 (SAS Institute, Inc., Cary, NC), based on the SAS codes provided by van Houwelingen et al. (Statistics in Medicine, 21: 589-624)

References to the Statistics:

Van Houwelingen H.C., Arends L.R., and Stijnen T. (2002). Advanced methods in meta-analysis: multivariate approach and meta-regression. Stat. Med. 21: 589-624.

DerSimonian R. and Laird N. (1986). Meta-analysis in clinical trials. Control. Clin. Trials. 7: 177-188.

Begg C.B. and Mazumdar M. (1994). Operating characteristics of a rank correlation test for publication bias. Biometrics 50: 1088-1101.

Egger M., Davey S.G., Schneider M., and Minder C. (1997). Bias in meta-analysis detected by a simple, graphical test. BMJ. 315: 629-634.

Online Supplement, Part 3. Results of the Automatic Search and the Manual Selection Process for the Inclusion of Publications in the Database.

Result of the selection process is shown separately in Online Supplementary Figure 1 (see below) for the add-on (left part) and monotherapy studies (right part).

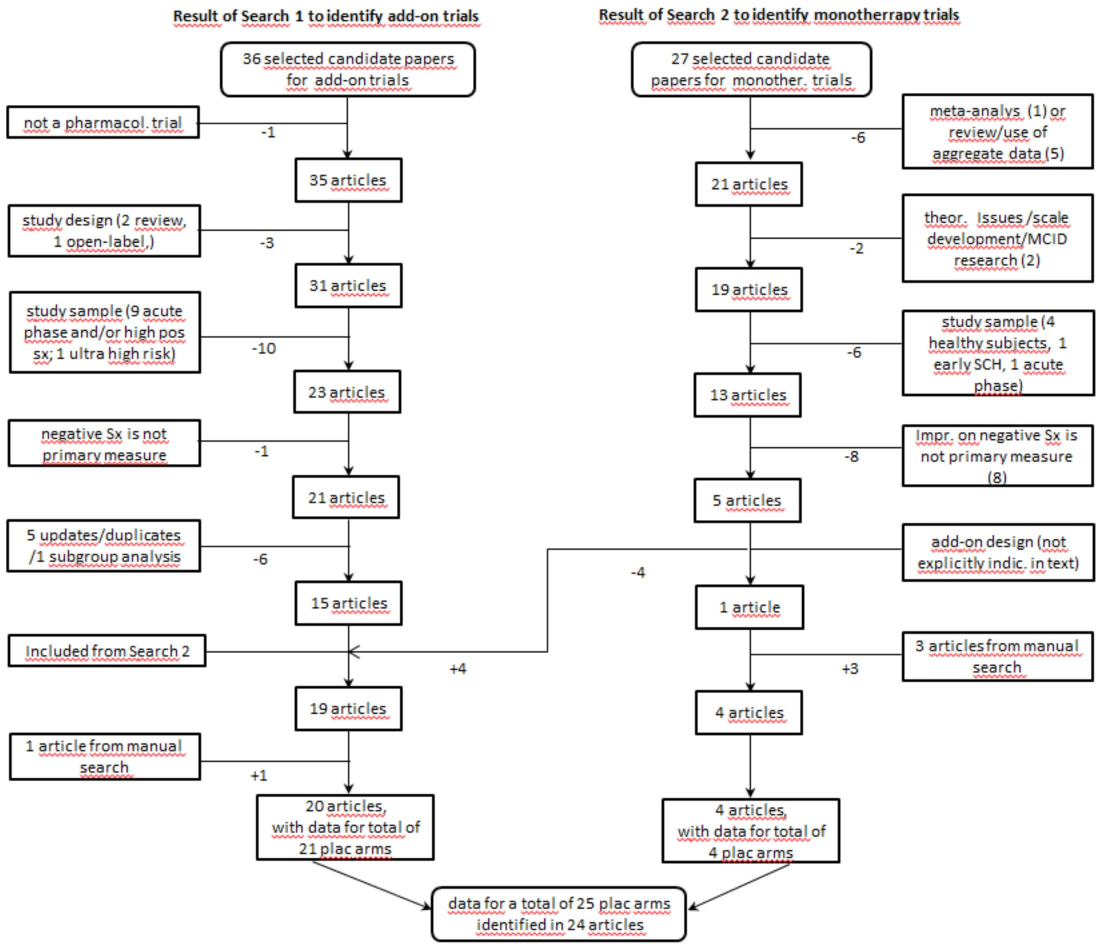

Online Supplementary Figure 1. Summary of the Automatic Search and the Manual Selection Process

(Abbreviations: negative Sx = negative symptoms; pos Sx = positive symptoms; plac = placebo)

As shown by the upper boxes in the Figure, the automatic search yielded 36 and 27 papers as candidates for inclusion into the add-on and monotherapy sets, respectively.

With respect to the add-on studies, the detailed evaluation of the full reports resulted in the exclusion of 21 papers. The exclusions occurred due to ineligibility of the study sample (n=10); updated reports becoming available for the same study (n=5); subgroup analysis (n=1); issues with the study design (n=3); and other reasons (not a pharmacological trial, n=1; the negative symptoms [NG] measure was not used as a primary end point, n=1). These exclusions led to the use of 15 papers from the automatic search. Additionally, five more papers were added to this set. Specifically, one paper was added on the basis of

manual search, while 4 others were included as a result of reclassification from the monotherapy set to the add-on set. In these cases of reclassification, none of the reports' respective key fields (titles, key words, abstract) explicitly indicated that the test treatment was applied as add-on medication (i.e., none of the relevant descriptors such as add-on/augmentation/adjunctive/supplement/supplementation/adjuvant were included in the key fields); however, the reading of the text revealed this information. As a result of the above described selection process, we identified 20 papers which reported with data for a total of 21 add-on studies (i.e., 21 samples receiving placebo).

With respect to monotherapy studies, the detailed evaluation of the full text reports resulted in the inclusion of only 1 paper from the automatic selection (n=27). The reasons for not qualifying for inclusion were that the publication was a review paper/meta-analytic summary using aggregate data (n=6); the report focused on theoretical/methodological issues on negative symptoms (n=2); the study sample did not represent the target population of the current investigation (n=6); or improvement on negative symptoms was not used as a primary endpoint (n=8). Additionally, 4 of the 26 studies from this set were reclassified as an add-on trial (see above). Three additional articles were added to the monotherapy set as a result of manual search, hence the monotherapy set comprised 4 publications. Finally, it should also be noted that 2 of the 4 monotherapy studies (*Moller HJ et al Pharmacopsychiatry 2004; 37: 270-278 and Lecrubier Y et al. Acta Psychiatr Scand 2006; 114: 319-327*) were included in Krause et al's meta-analysis of the drug-placebo difference *(Krause M., Zhu Y., Huhn M., Schneider-Thoma J., Bighelli I., Nikolakopoulou A., and Leucht S., 2018. Antipsychotic drugs for patients with schizophrenia and predominant or prominent negative symptoms: a systematic review and meta-analysis. Eur. Arch. Psychiatry. Clin. Neurosci. 268: 625-639)*; however, the effect size for the placebo response was not determined in that meta-analysis for these two studies.

The list below provides the set of publications that were considered for potential inclusion in the meta-analysis for the add-on and monotherapy trial set, respectively, based on the automatic (query) and manual search.

Final disposition status (Included/Not Included in the meta-analysis)for the ADD-ON trials. In case of exclusion, the reason of exclusion is indicated.

References

1. Ulrich S, Messer T. Review and meta-analysis of add-on tranylcypromine with antipsychotic drugs for the treatment of schizophrenia with predominant negative symptoms: a restoration of evidence. *Curr Med Res Opin* **2021**; 37: 1233-1248.

Disposition: Not Included. Reason of exclusion: Meta-analysis.

2. Hosseininasab M, Zarghami M, Mazhari S et al. Nanocurcumin as an Add-on to Antipsychotic Drugs for Treatment of Negative Symptoms in Patients With Chronic Schizophrenia: A Randomized, Double-Blind, Placebo-Controlled Study. *J Clin Psychopharmacol* **2021**; 41: 25-30.

Disposition: Included.

3. Strzelecki D, Kotlicka-Antczak M, Kaczmarek B, Jerczynska H, Wysokinski A. Serum levels of neuropeptide Y in patients with chronic schizophrenia during treatment augmentation with sarcosine (results of the double-blind randomized controlled PULSAR study). *Hum Psychopharmacol* **2021**; 36: e2770.

Disposition: Not Included. Reason of exclusion: Clinical data available in prior publication.

4. Schaefer M, Sarkar S, Theophil I, Leopold K, Heinz A, Gallinat J. Acute and Long-term Memantine Add-on Treatment to Risperidone Improves Cognitive Dysfunction in Patients with Acute and Chronic Schizophrenia. *Pharmacopsychiatry* **2020**; 53: 21-29.

Disposition: Not Included. Reason of exclusion: Negative symptoms are not primary measure.

5. Ding N, Li Z, Liu Z. Escitalopram augmentation improves negative symptoms of treatment resistant schizophrenia patients - A randomized controlled trial. *Neurosci Lett* **2018**; 681: 68-72.

Disposition: Not Included. Reason of exclusion: High level of positive symptoms.

6. Strzelecki D, Urban-Kowalczyk M, Wysokinski A. Serum levels of interleukin 6 in schizophrenic patients during treatment augmentation with sarcosine (results of the PULSAR study). *Hum Psychopharmacol* **2018**; 33: e2652.

Disposition: Included.

7. Bugarski-Kirola D, Blaettler T, Arango C et al. Bitopertin in Negative Symptoms of Schizophrenia-Results From the Phase III FlashLyte and DayLyte Studies. *Biol Psychiatry* **2017**; 82: 8-16.

Disposition: Included.

8. Hirayasu Y, Sato SI, Shuto N, Nakano M, Higuchi T. Efficacy and Safety of Bitopertin in Patients with Schizophrenia and Predominant Negative Symptoms: Subgroup Analysis of Japanese Patients from the Global Randomized Phase 2 Trial. *Psychiatry Investig* **2017**; 14: 63-73.

Disposition: Not Included. Reason of exclusion: Subgroup analysis of a full dataset.

9. Dunayevich E, Buchanan RW, Chen CY et al. Efficacy and safety of the glycine transporter type-1 inhibitor AMG 747 for the treatment of negative symptoms associated with schizophrenia. *Schizophr Res* **2017**; 182: 90-97.

Disposition: Included.

10. Strzelecki D, Kaluzynska O, Szyburska J, Wysokinski A. MMP-9 Serum Levels in Schizophrenic Patients during Treatment Augmentation with Sarcosine (Results of the PULSAR Study). *Int J Mol Sci* **2016**; 17.

Disposition: Not Included. Reason of exclusion: Clinical data available from a later publication from the same dataset.

11. Strzelecki D, Kaluzynska O, Wysokinski A. BDNF serum levels in schizophrenic patients during treatment augmentation with sarcosine (results of the PULSAR study). *Psychiatry Res* **2016**; 242: 54-60.

Disposition: Not Included. Reason of exclusion: Clinical data available from a later publication from the same dataset.

12. Barnes TR, Leeson VC, Paton C et al. Antidepressant Controlled Trial For Negative Symptoms In Schizophrenia (ACTIONS): a double-blind, placebo-controlled, randomised clinical trial. *Health Technol Assess* **2016**; 20: 1-46.

Disposition: Included.

13. Nikbakhat MR, Arabzadeh S, Zeinoddini A et al. Duloxetine Add-On to Risperidone for Treatment of Negative Symptoms in Patients with Stable Schizophrenia: Randomized Double-Blind Placebo-Controlled Study. *Pharmacopsychiatry* **2016**; 49: 162-169.

Disposition: Not Included. Reason of exclusion: Study population. High positive and general symptom scores.

14. Shoja SS, Akbari S. Intractability of Deficit Syndrome of Schizophrenia Against Adjunctive Modafinil. *J Clin Psychopharmacol* **2016**; 36: 45-49.

Disposition: Not Included. Reason of t of exclusion: Study population. Not predominant or prominent negative symptoms.

15. Usall J, Huerta-Ramos E, Labad J et al. Raloxifene as an Adjunctive Treatment for Postmenopausal Women With Schizophrenia: A 24-Week Double-Blind, Randomized, Parallel, Placebo-Controlled Trial. *Schizophr Bull* **2016**; 42: 309-317.

Disposition: Included.

16. Shoja SS, Jafarabad MS, Azizi R. Amelioration of deficit syndrome of schizophrenia by norepinephrine reuptake inhibitor. *Ther Adv Psychopharmacol* **2015**; 5: 263-270.

Disposition: Not Included. Reason of exclusion: Study population.

17. Strzelecki D, Podgorski M, Kaluzynska O et al. Supplementation of antipsychotic treatment with sarcosine - GlyT1 inhibitor - causes changes of glutamatergic (1)NMR spectroscopy parameters in the left hippocampus in patients with stable schizophrenia. *Neurosci Lett* **2015**; 606: 7-12.

Disposition: Included in the analysis for comparison purpuses with previous meta-analysis.

18. Strzelecki D, Podgorski M, Kaluzynska O et al. Supplementation of Antipsychotic Treatment with the Amino Acid Sarcosine Influences Proton Magnetic Resonance Spectroscopy Parameters in Left Frontal White Matter in Patients with Schizophrenia. *Nutrients* **2015**; 7: 8767-8782.

Disposition: Not Included. Reason of exclusion: Updated cinical data are available in later publication.

19. Amminger GP, Mechelli A, Rice S et al. Predictors of treatment response in young people at ultra-high risk for psychosis who received long-chain omega-3 fatty acids. *Transl Psychiatry* **2015**; 5: e495.

Disposition: Not Included. Reason of exclusion: Study population (high risk subjects).

20. Veerman SR, Schulte PF, Begemann MJ, de HL. Non-glutamatergic clozapine augmentation strategies: a review and meta-analysis. *Pharmacopsychiatry* **2014**; 47: 231-238.

Disposition: Not Included. Reason of exclusion: Meta-analysis.

21. Schoemaker JH, Jansen WT, Schipper J, Szegedi A. The selective glycine uptake inhibitor org 25935 as an adjunctive treatment to atypical antipsychotics in predominant persistent negative symptoms of schizophrenia: results from the GIANT trial. *J Clin Psychopharmacol* **2014**; 34: 190-198.

Disposition: Included.

22. Stauffer VL, Millen BA, Andersen S et al. Pomaglumetad methionil: no significant difference as an adjunctive treatment for patients with prominent negative symptoms of schizophrenia compared to placebo. *Schizophr Res* **2013**; 150: 434-441.

Disposition: Included.

23. Hinkelmann K, Yassouridis A, Kellner M, Jahn H, Wiedemann K, Raedler TJ. No effects of antidepressants on negative symptoms in schizophrenia. *J Clin Psychopharmacol* **2013**; 33: 686-690.

Disposition: Included.

24. Prikryl R, Ustohal L, Prikrylova KH et al. A detailed analysis of the effect of repetitive transcranial magnetic stimulation on negative symptoms of schizophrenia: a double-blind trial. *Schizophr Res* **2013**; 149: 167-173.

Disposition: Not Included. Reason of exclusion: Not a pharmacological trial.

25. Lasser RA, Dirks B, Nasrallah H et al. Adjunctive lisdexamfetamine dimesylate therapy in adult outpatients with predominant negative symptoms of schizophrenia: open-label and randomized-withdrawal phases. *Neuropsychopharmacology* **2013**; 38: 2140-2149.

Disposition: Not Included. Reason of exclusion: Study design (open label).

26. Weiser M, Heresco-Levy U, Davidson M et al. A multicenter, add-on randomized controlled trial of low-dose d-serine for negative and cognitive symptoms of schizophrenia. *J Clin Psychiatry* **2012**; 73: e728-e734.

Disposition: Included.

27. Usall J, Huerta-Ramos E, Iniesta R et al. Raloxifene as an adjunctive treatment for postmenopausal women with schizophrenia: a double-blind, randomized, placebo-controlled trial. *J Clin Psychiatry* **2011**; 72: 1552-1557.

Disposition: Included.

28. Ghaleiha A, Noorbala AA, Farnaghi F, Hajiazim M, Akhondzadeh S. A double-blind, randomized, and placebo-controlled trial of buspirone added to risperidone in patients with chronic schizophrenia. *J Clin Psychopharmacol* **2010**; 30: 678-682.

Disposition: Not Included. Reason of exclusion: Study population, POS Sx > NEG Sx at baseline; NEG Sx is not primary measure.

29. Akhondzadeh S, Ghayyoumi R, Rezaei F et al. Sildenafil adjunctive therapy to risperidone in the treatment of the negative symptoms of schizophrenia: a double-blind randomized placebo-controlled trial. *Psychopharmacology (Berl)* **2011**; 213: 809-815.

Disposition: Not Included. Reason of exclusion: Study population (patients are in the active phase of their illness).

30. Abbasi SH, Behpournia H, Ghoreshi A et al. The effect of mirtazapine add on therapy to risperidone in the treatment of schizophrenia: a double-blind randomized placebo-controlled trial. *Schizophr Res* **2010**; 116: 101-106.

Disposition: Not Included. Reason of exclusion: Study population (patients are in the active phase of their illness).

31. Akhondzadeh S, Malek-Hosseini M, Ghoreishi A, Raznahan M, Rezazadeh SA. Effect of ritanserin, a 5HT2A/2C antagonist, on negative symptoms of schizophrenia: a double-blind randomized placebo-controlled study. *Prog Neuropsychopharmacol Biol Psychiatry* **2008**; 32: 1879-1883.

Disposition: Not Included. Reason of exclusion: Study population (patients are in the active phase of their illness).

32. Amiri A, Noorbala AA, Nejatisafa AA et al. Efficacy of selegiline add on therapy to risperidone in the treatment of the negative symptoms of schizophrenia: a double-blind randomized placebo-controlled study. *Hum Psychopharmacol* **2008**; 23: 79-86.

Disposition: Not Included. Reason of exclusion: Study population (patients are in the active phase of their illness).

33. Buchanan RW, Javitt DC, Marder SR et al. The Cognitive and Negative Symptoms in Schizophrenia Trial (CONSIST): the efficacy of glutamatergic agents for negative symptoms and cognitive impairments. *Am J Psychiatry* **2007**; 164: 1593-1602.

Disposition: Included.

34. Pierre JM, Peloian JH, Wirshing DA, Wirshing WC, Marder SR. A randomized, double-blind, placebo-controlled trial of modafinil for negative symptoms in schizophrenia. *J Clin Psychiatry* **2007**; 68: 705-710.

Disposition: Included.

35. Goff DC, Herz L, Posever T et al. A six-month, placebo-controlled trial of D-cycloserine co-administered with conventional antipsychotics in schizophrenia patients. *Psychopharmacology (Berl)* **2005**; 179: 144-150.

Disposition: Included.

36. Strous RD, Maayan R, Lapidus R et al. Dehydroepiandrosterone augmentation in the management of negative, depressive, and anxiety symptoms in schizophrenia. *Arch Gen Psychiatry* **2003**; 60: 133-141.

Disposition: Included.

37. Bugarski-Kirola D, Arango C, Fava M et al. Pimavanserin for negative symptoms of schizophrenia: results from the ADVANCE phase 2 randomised, placebo-controlled trial in North America and Europe. *Lancet Psychiatry* **2022**; 9: 46-58.

Disposition: Included. (Reclassified here from monotherapy search.)

38. Buchanan RW, Weiner E, Kelly DL et al. Rasagiline in the Treatment of the Persistent Negative Symptoms of Schizophrenia. *Schizophr Bull* **2015**; 41: 900-908.

Disposition: Included. (Reclassified here from monotherapy search.)

39. Umbricht D, Alberati D, Martin-Facklam M et al. Effect of bitopertin, a glycine reuptake inhibitor, on negative symptoms of schizophrenia: a randomized, double-blind, proof-of-concept study. *JAMA Psychiatry* **2014**; 71: 637-646.

Disposition: Included. (Reclassified here from monotherapy search.)

40. Duncan EJ, Szilagyi S, Schwartz MP et al. Effects of D-cycloserine on negative symptoms in schizophrenia. *Schizophr Res* **2004**; 71: 239-248.

Disposition: Included. (Reclassified here from monotherapy search.)

41. Hill M, Shannahan K, Jasinski S et al. Folate supplementation in schizophrenia: a possible role for MTHFR genotype. *Schizophr Res* **2011**; 127: 41-45.

Disposition: Included. (Identified by manual search.)

Final disposition status (Included/Not Included in the meta-analysis)for the MONOTHERAPY trials. In case of exclusion, the reason of exclusion is indicated

References

1. Bugarski-Kirola D, Arango C, Fava M et al. Pimavanserin for negative symptoms of schizophrenia: results from the ADVANCE phase 2 randomised, placebo-controlled trial in North America and Europe. *Lancet Psychiatry* **2022**; 9**:** 46-58.

Disposition: Not Included. Reason of exclusion: Reclassified to add-on dataset.

2. Bulubas L, Goerigk S, Gomes JS et al. Cognitive outcomes after tDCS in schizophrenia patients with prominent negative symptoms: Results from the placebo-controlled STARTS trial. *Schizophr Res* **2021**; 235**:** 44-51.

Disposition: Not Included. Reason of exclusion: Negative symptoms are not primary measure.

3. Laszlovszky I, Barabassy A, Nemeth G. Cariprazine, A Broad-Spectrum Antipsychotic for the Treatment of Schizophrenia: Pharmacology, Efficacy, and Safety. *Adv Ther* **2021**; 38**:** 3652-3673.

Disposition: Not Included. Reason of exclusion: Review/use of aggregate data.

4. Daniel DG, Kott A, Saoud J et al. Do Patterns of Instability or Severity of Psychopathology During Screening Predict Relapse in Schizophrenic Outpatient Subjects with Moderate to Severe Negative Symptoms Assigned to Placebo? *Innov Clin Neurosci* **2020**; 17**:** 27-29.

Disposition: Not Included. Reason of exclusion: Negative symptom improvement is not primary measure.

5. Strauss GP, Zamani EF, Sayama H et al. Network Analysis Indicates That Avolition Is the Most Central Domain for the Successful Treatment of Negative Symptoms: Evidence From the Roluperidone Randomized Clinical Trial. *Schizophr Bull* **2020**; 46**:** 964-970.

Disposition: Not Included. Reason of exclusion: Negative symptom improvement is not primary measure.

6. Abram SV, De CL, Roach BJ et al. Oxytocin Enhances an Amygdala Circuit Associated With Negative Symptoms in Schizophrenia: A Single-Dose, Placebo-Controlled, Crossover, Randomized Control Trial. *Schizophr Bull* **2020**; 46**:** 661-669.

Disposition: Not Included. Reason of exclusion: Negative symptom improvement is not primary measure.

7. Misiak B, Bienkowski P, Samochowiec J. Cariprazine - a novel antipsychotic drug and its place in the treatment of schizophrenia. *Psychiatr Pol* **2018**; 52**:** 971-981.

Disposition: Not Included. Reason of exclusion: Review/use of aggregate data.

8. Earley W, Guo H, Daniel D et al. Efficacy of cariprazine on negative symptoms in patients with acute schizophrenia: A post hoc analysis of pooled data. *Schizophr Res* **2019**; 204**:** 282-288.

Disposition: Not Included. Reason of exclusion: Review/use of aggregate data.

9. Keefe RSE, Harvey PD, Khan A et al. Cognitive Effects of MIN-101 in Patients With Schizophrenia and Negative Symptoms: Results From a Randomized Controlled Trial. *J Clin Psychiatry* **2018**; 79.

Disposition: Not Included. Reason of exclusion: Negative symptom improvement is not primary measure.

10. Fraguas D, Diaz-Caneja CM, Pina-Camacho L, Umbricht D, Arango C. Predictors of Placebo Response in Pharmacological Clinical Trials of Negative Symptoms in Schizophrenia: A Meta-regression Analysis. *Schizophr Bull* **2019**; 45**:** 57-68.

Disposition: Not Included. Reason of exclusion: Meta-analysis.

11. Buchanan RW, Weiner E, Kelly DL et al. Rasagiline in the Treatment of the Persistent Negative Symptoms of Schizophrenia. *Schizophr Bull* **2015**; 41**:** 900-908.

Disposition: Not Included. Reason of exclusion: Reclassified to add-on dataset.

12. Levine SZ, Leucht S. Treatment response heterogeneity in the predominant negative symptoms of schizophrenia: analysis of amisulpride vs placebo in three clinical trials. *Schizophr Res* **2014**; 156**:** 107-114.
Disposition: Not Included. Reason of exclusion: Use of aggregate data.
13. Umbricht D, Alberati D, Martin-Facklam M et al. Effect of bitopertin, a glycine reuptake inhibitor, on negative symptoms of schizophrenia: a randomized, double-blind, proof-of-concept study. *JAMA Psychiatry* **2014**; 71**:** 637-646.
Disposition: Not Included. Reason of exclusion: Reclassified to add-on dataset.
14. Levine SZ, Leucht S. Identifying clinically meaningful symptom response cut-off values on the SANS in predominant negative symptoms. *Schizophr Res* **2013**; 145**:** 125-127.
Disposition: Not Included. Reason of exclusion: Deals with theoretical issues/scale development.
15. Levine SZ, Leucht S. Psychometric analysis in support of shortening the Scale for the Assessment of Negative Symptoms. *Eur Neuropsychopharmacol* **2013**; 23**:** 1051-1056.
Disposition: Not Included. Reason of exclusion: Deals with theoretical issues/scale development.
16. Levine SZ, Leucht S. Attaining and sustaining remission of predominant negative symptoms. *Schizophr Res* **2013**; 143**:** 60-64.
Disposition: Not Included. Reason of exclusion: Negative symptom improvement is not primary measure.
17. Yoon KS, Park TW, Yang JC et al. Different safety profiles of risperidone and paliperidone extended-release: a double-blind, placebo-controlled trial with healthy volunteers. *Hum Psychopharmacol* **2012**; 27**:** 305-314.
Disposition: Not Included. Reason of exclusion: Study population (healthy subjects).
18. Chaudhry IB, Hallak J, Husain N et al. Minocycline benefits negative symptoms in early schizophrenia: a randomised double-blind placebo-controlled clinical trial in patients on standard treatment. *J Psychopharmacol* **2012**; 26**:** 1185-1193.
Disposition: Not Included. Reason of exclusion: Study population (early schizophrenia).
19. Park CH, Park TW, Yang JC et al. No negative symptoms in healthy volunteers after single doses of amisulpride, aripiprazole, and haloperidol: a double-blind placebo-controlled trial. *Int Clin Psychopharmacol* **2012**; 27**:** 114-120.
Disposition: Not Included. Reason of exclusion: Study population (healthy subjects).
20. Chen ZH, Wang GH, Wang XP et al. Effect of Warm-Supplementing Kidney Yang (WSKY) added to risperidone on quality of life in patients with schizophrenia: a randomized controlled trial. *Clin Rehabil* **2009**; 23**:** 963-972.
Disposition: Not Included. Reason of exclusion: Negative symptom improvement is not primary measure.
21. Canuso CM, Bossie CA, Turkoz I, Alphs L. Paliperidone extended-release for schizophrenia: effects on symptoms and functioning in acutely ill patients with negative symptoms. *Schizophr Res* **2009**; 113**:** 56-64.
Disposition: Not Included. Reason of exclusion: Study population (patients are in acute phase).
22. Hunter MD, Ganesan V, Wilkinson ID, Spence SA. Impact of modafinil on prefrontal executive function in schizophrenia. *Am J Psychiatry* **2006**; 163**:** 2184-2186.
Disposition: Not Included. Reason of exclusion: Negative symptom improvement is not primary measure.
23. Lecrubier Y, Quintin P, Bouhassira M, Perrin E, Lancrenon S. The treatment of negative symptoms and deficit states of chronic schizophrenia: olanzapine compared to amisulpride and placebo in a 6-month double-blind controlled clinical trial. *Acta Psychiatr Scand* **2006**; 114**:** 319-327.
Disposition: Included.
24. Artaloytia JF, Arango C, Lahti A et al. Negative signs and symptoms secondary to antipsychotics: a double-blind, randomized trial of a single dose of placebo, haloperidol, and risperidone in healthy volunteers. *Am J Psychiatry* **2006**; 163**:** 488-493.
Disposition: Not Included. Reason of exclusion: Study population (healthy subjects).
25. Duncan EJ, Szilagyi S, Schwartz MP et al. Effects of D-cycloserine on negative symptoms in schizophrenia. *Schizophr Res* **2004**; 71**:** 239-248.
Disposition: Not Included. Reason of exclusion: Reclassified to add-on dataset.
26. Abel KM, Allin MP, Kucharska-Pietura K et al. Ketamine and fMRI BOLD signal: distinguishing between effects mediated by change in blood flow versus change in cognitive state. *Hum Brain Mapp* **2003**; 18**:** 135-145.
Disposition: Not Included. Reason of exclusion: Study population (healthy subjects).
27. Curran MP, Perry CM. Spotlight on amisulpride in schizophrenia. *CNS Drugs* **2002**; 16**:** 207-211.
Disposition: Not Included. Reason of exclusion: Review/use of aggregate data.

28. Moller HJ, Riedel M, Muller N, Fischer W, Kohnen R. Zotepine versus placebo in the treatment of schizophrenic patients with stable primary negative symptoms: a randomized double-blind multicenter trial. *Pharmacopsychiatry* **2004**; 37: 270-278.
Disposition: Included. (Identified by manual search).
29. Davidson M, Saoud J, Staner C et al. Efficacy and Safety of MIN-101: A 12-Week Randomized, Double-Blind, Placebo-Controlled Trial of a New Drug in Development for the Treatment of Negative Symptoms in Schizophrenia. *Am J Psychiatry* **2017**; 174: 1195-1202.
Disposition: Included. (Identified by manual search).
30. Davidson M, Saoud J, Staner C et al. Efficacy and Safety of Roluperidone for the Treatment of Negative Symptoms of Schizophrenia. *Schizophr Bull* **2022**. Feb. 25. 6536645 [pii];10.1093/schbul/sbac013 [doi]
Disposition: Included. (Identified by manual search).

Online Supplement, Part 4. List of the final set of publications included in the meta-analysis.

Final set of add-on trial publications included in the current meta-analysis

References

1. Buchanan RW, Javitt DC, Marder SR et al. The Cognitive and Negative Symptoms in Schizophrenia Trial (CONSIST): the efficacy of glutamatergic agents for negative symptoms and cognitive impairments. *Am J Psychiatry* **2007**; 164: 1593-1602.
2. Buchanan RW, Weiner E, Kelly DL et al. Rasagiline in the Treatment of the Persistent Negative Symptoms of Schizophrenia. *Schizophr Bull* **2015**; 41: 900-908.
3. Bugarski-Kirola D, Blaettler T, Arango C et al. Bitopertin in Negative Symptoms of Schizophrenia-Results From the Phase III FlashLyte and DayLyte Studies. *Biol Psychiatry* **2017**; 82: 8-16.
4. Dunayevich E, Buchanan RW, Chen CY et al. Efficacy and safety of the glycine transporter type-1 inhibitor AMG 747 for the treatment of negative symptoms associated with schizophrenia. *Schizophr Res* **2017**; 182: 90-97.
5. Duncan EJ, Szilagyi S, Schwartz MP et al. Effects of D-cycloserine on negative symptoms in schizophrenia. *Schizophr Res* **2004**; 71: 239-248.
6. Goff DC, Herz L, Posever T et al. A six-month, placebo-controlled trial of D-cycloserine co-administered with conventional antipsychotics in schizophrenia patients. *Psychopharmacology (Berl)* **2005**; 179: 144-150.
7. Hill M, Shannahan K, Jasinski S et al. Folate supplementation in schizophrenia: a possible role for MTHFR genotype. *Schizophr Res* **2011**; 127: 41-45.
8. Hinkelmann K, Yassouridis A, Kellner M, Jahn H, Wiedemann K, Raedler TJ. No effects of antidepressants on negative symptoms in schizophrenia. *J Clin Psychopharmacol* **2013**; 33: 686-690.
9. Pierre JM, Peloian JH, Wirshing DA, Wirshing WC, Marder SR. A randomized, double-blind, placebo-controlled trial of modafinil for negative symptoms in schizophrenia. *J Clin Psychiatry* **2007**; 68: 705-710.
10. Schoemaker JH, Jansen WT, Schipper J, Szegedi A. The selective glycine uptake inhibitor org 25935 as an adjunctive treatment to atypical antipsychotics in predominant persistent negative symptoms of schizophrenia: results from the GIANT trial. *J Clin Psychopharmacol* **2014**; 34: 190-198.
11. Stauffer VL, Millen BA, Andersen S et al. Pomaglumetad methionil: no significant difference as an adjunctive treatment for patients with prominent negative symptoms of schizophrenia compared to placebo. *Schizophr Res* **2013**; 150: 434-441.

12. Strous RD, Maayan R, Lapidus R et al. Dehydroepiandrosterone augmentation in the management of negative, depressive, and anxiety symptoms in schizophrenia. *Arch Gen Psychiatry* **2003**; 60: 133-141.
13. Strzelecki D, Podgorski M, Kaluzynska O et al. Supplementation of Antipsychotic Treatment with the Amino Acid Sarcosine Influences Proton Magnetic Resonance Spectroscopy Parameters in Left Frontal White Matter in Patients with Schizophrenia. *Nutrients* **2015**; 7: 8767-8782.
14. Umbricht D, Alberati D, Martin-Facklam M et al. Effect of bitopertin, a glycine reuptake inhibitor, on negative symptoms of schizophrenia: a randomized, double-blind, proof-of-concept study. *JAMA Psychiatry* **2014**; 71: 637-646.
15. Usall J, Huerta-Ramos E, Iniesta R et al. Raloxifene as an adjunctive treatment for postmenopausal women with schizophrenia: a double-blind, randomized, placebo-controlled trial. *J Clin Psychiatry* **2011**; 72: 1552-1557.
16. Usall J, Huerta-Ramos E, Labad J et al. Raloxifene as an Adjunctive Treatment for Postmenopausal Women With Schizophrenia: A 24-Week Double-Blind, Randomized, Parallel, Placebo-Controlled Trial. *Schizophr Bull* **2016**; 42: 309-317.
17. Weiser M, Heresco-Levy U, Davidson M et al. A multicenter, add-on randomized controlled trial of low-dose d-serine for negative and cognitive symptoms of schizophrenia. *J Clin Psychiatry* **2012**; 73: e728-e734.
18. Strzelecki D, Urban-Kowalczyk M, Wysokinski A. Serum levels of TNF-alpha in patients with chronic schizophrenia during treatment augmentation with sarcosine (results of the PULSAR study). *Psychiatry Res* **2018**; 268: 447-453.
19. Barnes TR, Leeson VC, Paton C et al. Antidepressant Controlled Trial For Negative Symptoms In Schizophrenia (ACTIONS): a double-blind, placebo-controlled, randomised clinical trial. *Health Technol Assess* **2016**; 20: 1-46.
20. Bugarski-Kirola D, Arango C, Fava M et al. Pimavanserin for negative symptoms of schizophrenia: results from the ADVANCE phase 2 randomised, placebo-controlled trial in North America and Europe. *Lancet Psychiatry* **2022**; 9: 46-58.
21. Hosseininasab M, Zarghami M, Mazhari S et al. Nanocurcumin as an Add-on to Antipsychotic Drugs for Treatment of Negative Symptoms in Patients With Chronic Schizophrenia: A Randomized, Double-Blind, Placebo-Controlled Study. *J Clin Psychopharmacol* **2021**; 41: 25-30.

Final set of monotherapy trial publications included in the meta-analysis
References

1. Moller HJ, Riedel M, Muller N, Fischer W, Kohnen R. Zotepine versus placebo in the treatment of schizophrenic patients with stable primary negative symptoms: a randomized double-blind multicenter trial. *Pharmacopsychiatry* **2004**; 37: 270-278.
2. Lecrubier Y, Quintin P, Bouhassira M, Perrin E, Lancrenon S. The treatment of negative symptoms and deficit states of chronic schizophrenia: olanzapine compared to amisulpride and placebo in a 6-month double-blind controlled clinical trial. *Acta Psychiatr Scand* **2006**; 114: 319-327.
3. Davidson M, Saoud J, Staner C et al. Efficacy and Safety of MIN-101: A 12-Week Randomized, Double-Blind, Placebo-Controlled Trial of a New Drug in Development for the Treatment of Negative Symptoms in Schizophrenia. *Am J Psychiatry* **2017**; 174: 1195-1202.
4. Davidson M, Saoud J, Staner C et al. Efficacy and Safety of Roluperidone for the Treatment of Negative Symptoms of Schizophrenia. *Schizophr Bull* **2022**.

Online Supplement, Part 5. Description of the negative symptom measures used in each study & the computational details for the severity of negative symptoms at baseline.

Online Table 1 (column 3) describes the negative (NEG) symptom measure that was used as a primary endpoint for the negative symptoms (NS) in each of the individual studies. Columns 4 and 5 provide the data used for the computation of baseline severity% (in column 6) in the current analysis. Please note that our computation of baseline severity differed from that of Fraguas et al *(Fraguas D., Diaz-Caneja C.M., Pina-Camacho L., Umbricht D., and Arango C. (2019). Predictors of Placebo Response in Pharmacological Clinical Trials of Negative Symptoms in Schizophrenia: A Meta-regression Analysis*. *Schizophr. Bull. 45: 57-68)*, who expressed baseline severity as a fraction of the range between zero and the maximum score. Specifically, they divided the baseline value on the NS scale of interest by the maximum of the scale. This approach is incorrect when the scale's minimum value (when no symptoms are observable) is not at zero. In such case (e.g., for the PANSS, where for each individual item the scale's zero-point has a value of 1) a correction for the scale's zero-point is needed (i.e., subtraction of the scale's minimum from the maximum value for the computation). For example, for the PANSS negative subscale, which has 7 items with an item-score range of 1 through 7, the complete absence of symptoms at baseline would be associated with a subscale score of 7. Thus, although the true baseline severity score fraction should be at 0% in this case, the uncorrected formula, used in the prior meta-analysis, yields 14.3% (=100*(7/49)), as it expresses baseline severity as a fraction of the maximum without adjusting for the scale's actual zero-point. The table below indicates (in column 9) whether the computation of baseline in the current meta-analysis was different from that of Fraguas et al.'s meta-analysis due to the aforementioned problem, or for any other reason.

Online Table 1. Measures of negative symptoms and computation of baseline severity for the studies included in the current meta-analysis

1 Studies[a]	2 Study-type	3 NEG symptom measure	4 NEG symp-tom severity at baseline	5 NEG symp-tom scale max	6 %NEG (base-line severity current meta)	7 NEG scale max (prior meta)	8 %NEG (base-line severity prior meta)	9 Baseline compu-tation differs in prior analysis[c]	Comment
Buchanan, 2007	add-on	modif. SANS average item score	2.3	5	46	5	46	0	Average item score used based on 1982 version of SANS, ATTN not included.
Buchanan, 2015	add-on	modif. SANS total	33.5	85	39.4	80	42	1	Modif. SANS was used; ATTN was not included, but 1 extra item was added, overlooked in prior analysis.
Bugarski Kirola, 2017 DayLyte study	add-on	PANSS NEG factor score (Marder factor)	20.8	42	49.5	42	50	0	Original paper used corrected PANSS, no need to subtract the min. score of 7.
Bugarski Kirola, 2017 FlashLyte study	add-on	PANSS NEG factor score (Marder	20.1	42	47.9	42	48	0	Original paper used corrected PANSS, no need to subtract the min. score of 7.

		factor)							
Dunajevich, 2017	add-on	NSA 16 total	58.8	96	61.3	96	61	0	-
Duncan, 2004	add-on	SANS total	70	95	73.7	120	58	1	Reference in original paper is to 1989 publication on SANS, which has 19 items, with a total score=95.
Goff, 2005	add-on	modif. SANS total	51.3	95	54	120	43	1	SANS without inappropriate affect and subjective items; ATTN is included (publication refers to mod. SANSS scale; these was overlooked in prior analysis.
Hill, 2011	add-on	modif. SANS total	45.8	90	50.9	90	51	0	Modified SANS was used, without ATTN items.
Hinkelmann, 2013	add-on	PANSS NEG subscale	26.7	49	46.9	49	54	1	Subtraction of the min. scale value of 7 needed, original paper used uncorrected PANSS subscale.
Pierre, 2007	add-on	modif. SANS total	38.5	90	42.8	80	48	0	SANS 18 item modif. version used, without ATTN item (as stated in the original paper).
Shoemaker, 2014	add-on	modif. SANS total	63.9	110	58.1	120	53	1	Original paper indicates that composite score was computed based on items 1 through 22; this was overlooked in prior meta-analysis.
Stouffer, 2013	add-on	NSA 16 total	59.6	96	62.1	96	62	0	-
Strous, 2003	add-on	modif. SANS total	41	95	43.2	n/a	n/a	n/a	Modif. SANS version (1989); baseline value was extracted from figure in original paper for this meta-analysis (missing value was used in prior meta-analysis).
Umbricht, 2014	add-on	PANSS NEG factor score (Marder factor)	25.9	49	45.0	49	53	1	Subtraction of the min. scale value of 7 needed, original paper used uncorrected PANSS subscale.
Usall, 2011	add-on	PANSS NEG subscale	21.63	49	34.8	n/a	n/a	1	Prior meta-analysis indicated baseline as missing; subtraction of the min scale value of needed.
Usall, 2016	add-on	PANSS NEGG subscale	22.81	49	37.6	49	47	1	Subtraction of the min. scale value of 7 needed, original paper used uncorrected PANSS subscale.
Weiser, 2012	add-on	modif. SANS total	58.9	90	65.4	120	49	1	Modif. SANS version used, which did not include the ATTN item.
Strzelecki, 2018[b]	add-on	PANSS NEG subscale	26.1	49	45.5	n/a	n/a	n/a	Subtraction of the min. scale value of 7 needed, original paper used uncorrected PANSS subscale.
Barnes, 2016	add-on	PANSS NEG subscale	25.7	49	44.5	n/a	n/a	n/a	Subtraction of the min. scale value of 7 needed, original paper used uncorrected PANSS subscale.
Bugarski-Kirola, 2022	add-on	NSA 16 total	61	96	63.5	n/a	n/a	n/a	-
Hosseininasab, 2021	add-on	PANSS NEG subscale	22.8	49	37.6	n/a	n/a	n/a	Subtraction of the min. scale value of 7 needed, original paper used uncorrected PANSS subscale.
Möller, 2004	monoth.	PANSS NEG subscale	27.5	49	48.8	n/a	n/a	n/a	Subtraction of the min scale value of 7 needed, original paper used uncorrected PANSS subscale.
Lecrubier, 2006	monoth.	modif. SANS, summary score	15.8	20	79.0	n/a	n/a	n/a	Summary SANS score (sum of global scores), excluding ATTN.
Davidson, 2017	monoth.	PANSS NEG factor (Pentagonal model)	31.5	70	35.8	n/a	n/a	n/a	Subtraction of the min. scale value of 7 needed, original paper used uncorrected PANSS subscale.

Davidson, 2022	monoth.	PANSS NEG factor score (Marder factor)	24	49	40.5	n/a	n/a	n/a	Subtraction of the min. scale value of 7 needed, original paper used uncorrected PANSS subscale.

Notes:

Abbreviations: SANS = Scale for the Assessment of Negative Symptoms; PANSS = Positive and Negative Symptoms Scale; NSA 16 = Negative Symptom Assessment-16 scale; NEG = negative symptoms; monoth.= monotherapy; modif. SANS = modified version of the original SANS scale; ATTN = attention items; min.= minimum; original paper = refers to the publication which provided the source data for the trial included in the current meta-analysis

[a]: Data from 25 studies, reported in 24 publications, were used in the current meta-analysis. Note: One publication (Bugarski Kirola, 2017) reported data from two studies (DayLyte & FlashLyte).

[b]: Clinical data for the PULSAR study were extracted for the current meta-analysis from Strzelecki, 2018 (Strzelecki D et al. Psychiatry Res 2018; 268: 447-453). As the prior meta-analysis by Fraguas et al (Schizophr. Bull. 2019, 45: 57-68) covered a time period before 2018, it extracted data from an earlier report available for this study (Strzelecki D et al. Nutrients 2015; 7: 8767-8782).

[c]: Computation/determination of baseline severity (%) on negative symptoms was different in the current meta-analysis from the prior one by Fraguas et al. for the following studies (listed below according to the specific reason):

- Hinkelmann 2013, Umbricht 2014, Usall 2016: Subtraction of the min. scale value (i.e., 7) was needed to correct for the condition that the subscale's minimum value is non-zero (i.e., 7); this was not done in prior meta-analysis.
- Goff, 2005: The SANS rating did not include inappropriate affect and subjective items while ATTN was included; adjustment was done only for one of these two changes in prior meta-analysis.
- Shoemaker, 2014: the composite score was computed based on items 1 through 22 as indicated in the original paper; this was overlooked in prior meta-analysis.
- Usall, 2011: prior meta-analysis indicated that the baseline value was missing; it was, however, available in the original paper.
- Weiser, 2012: the modified SANS was used, which did not include ATTN; this was overlooked in prior meta-analysis.
- Strous, 2003: prior meta-analysis indicated that the baseline value was missing; for the current meta-analysis the value was determined from the pertinent figure in the original paper.
- Additional note for Umbricht, 2014: PANSS negative factor (i.e., Marder factor) score was used instead of the PANSS negative symptom subscale (which was indicated in prior meta-analysis).

Online Supplement, Part 6. Basic Demographical and Clinical Characteristics of the Study Sample Included in the Publications.

The patient population in the placebo arms of the included studies was chronically ill, with an average age of 44.0 years (SD=6.9) and a mean duration of illness of 20.1 years (SD=8.1). Females were underrepresented in the samples (mean female %=39.6, SD=26.2), especially if we consider the median value (Q50=34.2% females). In terms of baseline severity, the study samples were located in average somewhat higher than the midpoint in the range between the minimum and maximum of the negative symptom scale used in the study (mean=53.4%, SD=9.5%). The SANS scale for the negative symptom rating was applied 10 of 25 studies, while in 12 and 3 of the included studies the PANSS and NSA-16 scales were used, respectively. Of the 12 studies that applied the PANSS, 7 used the negative symptom subscale, while 5 used a negative symptom factor (4 employed the Marder factor; and 1 employed the negative factor from the PANSS's pentagonal factor structure). Details regarding the computation of the baseline severity of the negative symptoms for each study and the further description of the negative symptom measures used in the individual studies included in the meta-analysis are summarized above in Online Supplementary Table 1.

Online Table 2. Basic descriptive data on the placebo samples and clinical trial characteristics for the included studies.

**Characteristic**			**Sample Statistics**			
	N of Studies	Mean	SD	Q25[c]	Q50[c]	Q75[c]
Average age (years)	25	44.0	6.9	39.0	42.3	46.5
Duration of illness (years)	19	20.1	8.1	12.8	18.8	25.4
Female (%)	24	39.6	26.2	28.6	34.2	46.8
Baseline severity on NEG, %[a]	25	53.4	9.5	46.5	52.4	58.1
Placebo N	25	63.7	62.1	17.0	34.0	79.0
Total N	25	175.5	175.2	54.0	79.0	235.0
	N of Studies	N	%			
Test drug	25					
Add-on		21	84.0	-	-	-
Monotherapy		4	16.0	-	-	-
Single/Multi-center	25					
Single center		7	28.0	-	-	-
Multicenter		18	72.0	-	-	-
Trial duration	25					
Short-term (≤8 weeks)		6	24.0	-	-	-
Longer-term (>8 weeks)		19	76.0	-	-	-
Active (Test) / Placebo ratio[b]	25					
1:1		15	60.0	-	-	-
2:1		8	32.0	-	-	-
3:1		2	8.0	-	-	-
Industry sponsorship	25					
No		14	56.0	-	-	-
Yes		11	44.0	-	-	-

[a]: Baseline severity on negative symptoms (NEG, %): expressed as a percent value of the baseline severity in relation to the minimum and maximum value of the scale, e.g., a raw score of 28 on the PANSS negative symptom subscale yields 50% severity at baseline since it is located exactly in the middle between the minimum (7) and maximum (49) of the scale.

[b]: The proportion of patients randomized to the active (test) compound as compared to placebo.
[c]: Depicted values represent the median (Q50), and the interquartile range (Q25=lower 25% quartile, Q75=upper 25% quartile).

Online Supplement, Part 7. Specific reasons of reassessment of effect size estimates from previous meta-analysis.

Online Supplement, Table 2 (see below) lists the reasons why the approximately five-fold inflation of the ES occurred in the previous meta-analysis *(Fraguas D., Diaz-Caneja C.M., Pina-Camacho L., Umbricht D., and Arango C. (2019). Predictors of Placebo Response in Pharmacological Clinical Trials of Negative Symptoms in Schizophrenia: A Meta-regression Analysis***.** *Schizophr. Bull. 45***:** *57-68)*. For each study included both in the current and former meta-analysis, the table indicates whether a reassessment of the estimate was needed; and if reassessment was needed, it describes its cause. Additionally, to evaluate the impact of the reassessment, the table juxtaposes the effect size estimates from the prior and current meta-analysis for each of the pertinent studies, and for the pooled effect size estimates. Major corrections are highlighted in the Table.

Online Supplement, Table 2. Placebo effect size estimates for add-on and monotherapy studies in prior and current meta-analysis of pharmacological trials of predominant or prominent negative symptoms

**Studies**[a]	**Study type**	**Study included in Fraguas et al. meta-analysis**[b]	**Study included in current meta-analysis**[c]	**Prior results Cohen's d effect size estimate from Fraguas et al.** d (SE) t p	**Reassessment (yes/no)) & reason**	**Current analysis Reassessed Cohen' d estimate** d (SE) t p
Buchanan, 2007	add-on	yes	yes	6.750 (0.676) 9.981 .000	**YES Major correction SE was used instead of SD**	0.936 (0.166) 5.629, .000
Buchanan, 2015	add-on	yes	yes	-0.143 (0.190) -0.752, .452	NO	-0.143 (0.190) -0.752, .452
Bugarski-Kirola, 2017, DayLyte Study[c]	add-on	yes	yes	11.753 (0.590) 19.906, .000	**YES Major correction SE was used instead of SD**	0.829 (0.082) 10.14, .000
Bugarski-Kirola, 2017, FlashLyte Study[c]	add-on	yes	yes	14.261 (0.735) 19.938, .000	**YES Major correction SE was used instead of SD**	1.035 (0.090) 11.51, .000
Dunayevich, 2017	add-on	yes	yes	4.917 (0.421) 11.691, .000	**YES Major correction SE was used instead of SD**	0.572 (0.125) 4.559, .000
Duncan, 2004	add-on	yes	yes	0.699 (0.322) 2.17, .03	YES sensitivity analysis	0.570 (0.311) 1.832, .067
Goff, 2005	add-on	yes	yes	0.593 (0.313) 1.896, .058	YES sensitivity analysis	0.552 (0.310) 1.782, .075

Hill, 2011	add-on	yes	yes	3.089 (0.642) 4.811, .000	**YES Major correction wrong column used for SD**	1.141 (0.343) 3.323, .001
Hinkelmann, 2013	add-on	yes	yes	1.083 (0.315) 3.440, .001	YES sensitivity analysis	0.902 (0.297) 3.043, .002
Pierre, 2007	add-on	yes	yes	0.297 (0.323) 0.920, .358	YES sensitivity analysis	0.243 (0.321) 0.758, .449
Schoemaker, 2014	add-on	yes	yes	0.994 (0.155) 6.402, .000	NO	0.994 (0.155) 6.402, .000
Stauffer, 2013	add-on	yes	yes	0.541 (0.118) 4.572, .000	YES estimate in source paper differs from meta-analysis est.	0.340 (0.114) 2.994, .003
Strous, 2003	add-on	yes	yes	0.201 (0.292) 0.690, .490	NO	0.201 (0.292) 0.689, .491
Strzelecki, 2015[d]	add-on	yes	no	0.060 (0.200) 0.300, .764	YES sensitivity analysis	0.049 (0.200) 0.245, .807
Umbricht, 2014	add-on	yes	yes	8.379 (0.769) 10.891, .000	**YES Major correction SE was used instead of SD**	1.073 (0.161) 6.676, .000
Usall, 2011	add-on	yes	yes	0.680 (0.269) 2.528, .011	NO	0.680 (0.269) 2.528, .011
Usall, 2016	add-on	yes	yes	1.806 (0.223) 4.874, .000	**YES Major correction SE was used instead of SD**	0.186 (0.178) 1.044, .297
Weiser, 2012	add-on	yes	yes	0.489 (0.107) 4.576, .000	YES sensitivity analysis	0.400 (0.105) 3.812, .000
**Pooled estimate, add-on**	**add-on studies from Fraguas et al.**			**2.909 (0.438) 6.642, .000**		**0.5925 (0.090) 6.560, .000**
Strzelecki, 2018[d]	add-on	no	yes	n/a	n/a	0.412 (0.190) 2.165, .030
Barnes, 2016	add-on	no	yes	n/a	n/a	0.3815 (0.183) 2.083, .037
Bugarski-Kirola, 2022	add-on	no	yes	n/a	n/a	0.893 (0.083) 10.728, .000
Hosseininasab, 2021	add-on	no	yes	n/a	n/a	-0.007 (0.186) -0.038, .970
**Pooled estimate, add-on for ALL studies**	**All add-on studies**	-	-	n/a	-	**0.5898 (0.077) 7.66, .000**
Möller, 2004	monoth.	no	yes	n/a	n/a	1.054 (0.195) 5.410, .000
Lecrubier, 2006	monoth.	no	yes	n/a	n/a	0.646 (0.189) 3.426, .001

Davidson, 2017[e]	monoth.	no	yes	3.255 (0.334) <0.001	YES Major correction SE was used instead of SD	0.366 (0.116) 3.151, 0.002
Davidson, 2022	monoth.	no	yes		n/a	0.785 (0.087) 9.000, .000
**Pooled estimate, monotherapy**	**All monoth. studies**	**-**	**-**	**n/a**	**n/a**	**0.6993 (0.167) 4.20, .000**
**Pooled estimate, all combined**	**All add-on & monoth.**	**-**	**-**	**n/a**	**n/a**	**0.6444 (0.091) 7.08, .000**

Notes:
Abbreviations: monoth.= monotherapy; SE = Standard Error; SD = Standard Deviation.

[a]: Fraguas et al's meta-analysis (Schizophr. Bull. 45: 57-68) analyzed data from 18 studies, which were reported in 17 publications. Note: One publication (Bugarski Kirola, 2017) reported data from two studies (DayLyte & FlashLyte).

[b]: Current meta-analysis analyzed data from 25 studies, which were reported in 24 publications.

[c]: One publication (Bugarski Kirola, 2017) reported data from two studies (DayLyte & FlashLyte).

[d]: Strzelecki, 2015 (Strzelecki D et al. Nutrients 2015; 7: 8767-8782) was used in the prior meta-analysis by Fraguas et al (Schizophr. Bull. 45: 57-68), but an updated version of the clinical data became available in Strzelecki, 2018 (Strzelecki D et al. Psychiatry Res 2018; 268: 447-453); the updated data were used in the current analyses.

[e]: Results of a monotherapy study (Davidson, 2017) were published before the publication of the paper by Fraguas et al. The placebo effect size for this study was then provided for the publication for comparative purposes with the meta-analytic estimates from the add-on studies (see Discussion in Fraguas et al', Schizophr. Bull. 45: 57-68). However, the Cohen's d value was erroneously computed as 3.255; the actual value is 0.366.
